# A spatial similarity of stereochemical environments formed by amino acid residues defines a common epitope of two non-homologous proteins

**DOI:** 10.1038/s41598-019-51350-2

**Published:** 2019-10-15

**Authors:** Kentaro Nakashima, Shintaro Iwashita, Takehiro Suzuki, Chieko Kato, Toshiyuki Kohno, Yasutomi Kamei, Motoki Sasaki, Osamu Urayama, Yoshiko Ohno-Iwashita, Naoshi Dohmae, Si-Young Song

**Affiliations:** 1 Institute of Neuroscience, Tokushima Bunri University, Kagawa, 769-2193 Japan; 2 Department of Pharmacy, Iwaki Meisei University, Fukushima, 970-8551 Japan; 3 Biomolecular Characterization Unit, RIKEN Center for Sustainable Resource Science, Saitama, 351-0198 Japan; 4 Department of Biochemistry, Kitasato University School of Medicine, Kanagawa, 242-0374 Japan; 5 Laboratory of Molecular Nutrition, Graduate School of Environmental and Life Science, Kyoto Prefectural University, Kyoto, 606-0823 Japan; 6 Department of Basic Veterinary Medicine, Obihiro University of Agriculture and Veterinary Medicine, Hokkaido, 080-8555 Japan; 7 Faculty of Health Sciences, Tsukuba International University, Ibaraki, 300-0051 Japan

**Keywords:** Immunochemistry, Proteins

## Abstract

It is critical for development of high-quality antibodies in research and diagnostics to predict accurately their cross-reactivities with “off-target” molecules, which potentially induce false results. Herein, we report a good example of such a cross-reactivity for an off-target due to a stereochemical environment of epitopes, which does not simply depend on amino acid sequences. We found that significant subpopulation of a polyclonal peptide antibody against Bcnt (Bucentaur) (anti-BCNT-C antibody) cross-reacted with a completely different protein, glutamine synthetase (GS), and identified four amino acids, GYFE, in its C-terminal region as the core amino acids for the cross-reaction. Consistent with this finding, the anti-BCNT-C antibody strongly recognized endogenously and exogenously expressed GS in tissues and cultured cells by Western blotting and immunohistochemistry. Furthermore, we elucidated that the cross-reaction is caused by a spatial similarity between the stereochemical environments formed by amino acid residues, including the GYFE of GS and the GYIE of Bcnt, rather than by their primary sequences. These results suggest it is critical to comprehensively analyze antibody interactions with target molecules including off-targets with special attention to the physicochemical environments of epitope-paratope interfaces to decrease the risk of false interpretations of results using antibodies in science and clinical applications.

## Introduction

Antibodies (Abs) are very powerful tools not only in basic research but also in clinical application of molecular targeted therapies for cancer^1–3^ and autoimmune diseases such as rheumatoid arthritis^4,5^. Antibody (Ab) drugs are a rapidly growing class of biopharmaceuticals and a large number of novel promising Abs such as nivolumab and pembrolizumab have been generated during the last few years. To efficiently develop high quality Abs for research use as well as Ab drugs and vaccines, it is critical not only to design immunogens that generate Abs with quite specific immunoreactivity for on-target molecules but also to predict more accurately cross-reactivities of Abs with “off-targets”, which potentially induce side effects in therapies using Abs and vaccines^6^. The amino acid sequences of epitopes have been considered to be a primary basis for specific immunoreactions of Abs with target molecules. However, in addition to Abs against proteins, those against nonprotein molecules, such as anti-DNA and anti-phospholipid Abs, are also well known. On the other hand, recent reports demonstrated that a multispecific monoclonal Ab recognizes completely different linear epitope sequences with high affinity^7^, and progress is proceeding toward development of therapies by intentionally using such a dual-specific Ab^8^. These examples are difficult to understand from a simple grasp of epitopes based on only their amino acid sequences. The epitopes are conventionally classified into two groups, linear or sequential and discontinuous or conformational^9,10^. Such a conventional classification based on amino acid sequences is useful to simply explain epitopes of proteins but insufficient to more accurately understand epitopes in antigen-Ab interactions such as the above-described examples because these examples indicate that Ab recognition of target molecules is based on the physicochemical environment of epitopes and does not necessarily depend on their linear amino acid sequences. Accordingly, it is necessary to have a more comprehensive understanding of Ab specificities on the basis of physicochemical features of epitopes. In this report, we present a didactic example to consider these points.

We identified the Bcnt (Bucentaur)/Cfdp1 (craniofacial developmental protein 1) protein family, which probably function as a component of chromatin remodeling complex by the study of the yeast ortholog Swc5^11^. Previously, we raised an Ab against a peptide of a C-terminal region of BCNT (called BCNT-C), EELAIHNRGKEGYIERKA, which is evolutionarily conserved from yeast to humans^12^. This anti-BCNT-C Ab detected doublet bands of 43/45 kDa by Western blot analysis of proteins extracted from bovine and rat brains. We tried to isolate them by immunoprecipitation and identify them by mass spectrometry. Surprisingly, we obtained the unexpected result that the 43 kDa protein is glutamine synthetase (GS: NP_001035564.1); EC 6.3.1.2, also known as γ-glutamate: ammonia ligase^13,14^, an enzyme entirely unrelated to Bcnt, which catalyzes the ATP-dependent condensation of glutamate and ammonia to form glutamine. The anti-BCNT-C Ab can specifically detect not only recombinant GS but also endogenous GS in rat brain. This quite specific reactivity of the Ab with an off-target molecule prompted us to seek a common epitope between GS and Bcnt to elucidate a chemical basis for the cross-reactivity of the anti-BCNT-C Ab with GS. We report here that the core amino acids of the common epitope causing the cross-reaction were identified as GYFE in GS and GYIE in Bcnt. Also, we discuss here the significance of physico- or stereochemical environments in epitope–paratope^10^ or protein–protein interactions^15^ and findings on the proteome-wide analyses of epitopes^9^.

## Results

### Detection of the 43 kDa protein by anti-BCNT-C Ab

**Figure 1:**
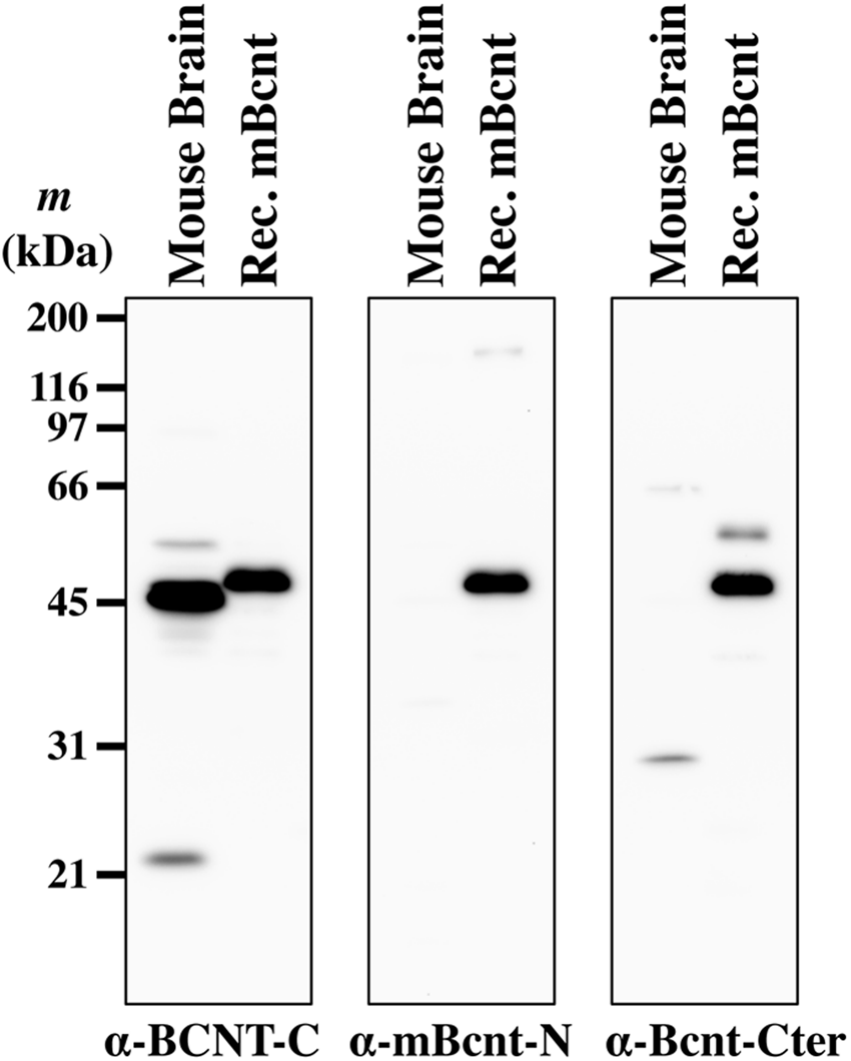
The 43 kDa protein as a major reactant in Western blotting with the anti-BCNT-C Ab. Western blotting was conducted with the polyclonal anti-BCNT-C, mBcnt-N and Bcnt-Cter Abs, as shown under each figure, using extracts of mouse brain and HEK293T cells transfected with mouse bcnt expression vector, as shown on the top of each lane. Note that a strong signal of the 43 kDa in mouse brain extracts was detected only by anti-BCNT-C Ab,while the recombinant mouse Bcnt protein was detected by all Abs.

Previously, we reported detection of strong doublet bands of 43/45 kDa by Western blot analysis of proteins extracted from bovine and rat brains with the anti-BCNT-C Ab^12^. This Ab reproducibly detected the 43/45 kDa proteins in mouse and bovine brain as well as the mouse Bcnt around 45 kDa exogenously expressed in HEK293T cells (Fig. 1 and see Supplementary Figs S1 and S2). To examine the specificity of the anti-BCNT-C Ab, we generated two other anti-Bcnt/Cfdp1 Abs using the C-terminus 10-mer peptide, which is evolutionarily conserved, (anti-Bcnt-Cter Ab) and the 14-mer peptide of the N-terminal region in mouse Bcnt (anti-mBcnt-N Ab), respectively, as different antigens from the anti-BCNT-C Ab (see Supplementary Table S1). The polyclonal anti-Bcnt-Cter Ab and the polyclonal anti-mBcnt-N Ab detected the recombinant Bcnt similarly as the anti-BCNT-C Ab, whereas they did not detect the 43/45 kDa proteins (Fig. 1). These results clearly indicate that 43/45 kDa proteins are specifically detected by only the anti-BCNT-C Ab, not the anti-Bcnt-Cter and the anti-mBcnt-N Ab. In addition to this discrepancy and different molecular sizes of these immunoreactants from the recombinant Bcnt (Fig. 1 and see Supplementary Fig. S2), their very high immunoreactivity prompted us to identify these proteins at the molecular level.

### Identification of the 43 kDa protein

**Figure 2:**
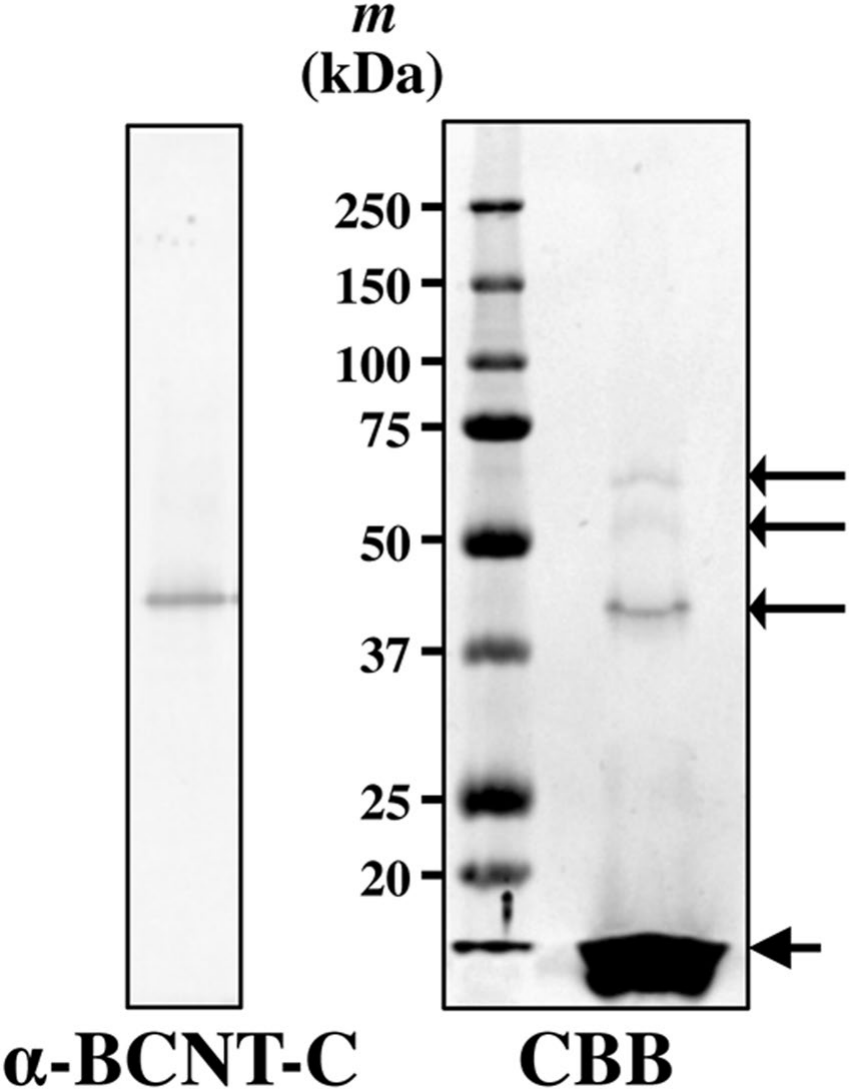
The isolated 43 kDa protein from bovine brain. The 43 kDa protein was isolated from bovine brain (see Supplementary Materials and Methods and Fig. S4). The eluted fractions from anti-BCNT-C Ab-linked agarose with the most positive immunoreactivities detected by a colorimetric method (left panel shows a representative fraction) were pooled, subjected to acetone precipitation, separated by SDS-PAGE and stained with Coomassie brilliant blue (right panel).

We found that we could immunoprecipitate the 43 kDa protein, but not the 45 kDa protein, from the extracts of bovine brain and rat olfactory bulb^16^ when the extracts were boiled once in SDS, whereas we failed using a conventional Radioimmunoprecipitation assay (RIPA) buffer (see Supplementary Fig. S3). Using this method, we isolated the 43 kDa protein from the extracts by ammonium sulfate precipitation, phenyl-sepharose chromatography (see Supplementary Fig. S4A), and immunoprecipitation using agarose beads coupled with the anti-BCNT-C Ab (see Supplementary Fig. S4B). Three broad Coomassie Brilliant Blue (CBB)-positive bands with a molecular mass of 43, 55 or 65 kDa, respectively, were detected on SDS-Polyacrylamide gel electrophoresis (SDS-PAGE) of immunoprecipitated proteins (Fig. 2). Protein mixtures contained in each separate band were subjected to LC-MS/MS analysis. Surprisingly, results from the analyses showed that glutamine synthetase (GS) was detected in all three bands, whereas no Bcnt fragment was detected in any analyzed band except for the GKEGYIER that was derived from the immunogen peptide used for elution of bound proteins from the affinity beads. In particular, GS having a calculated molecular mass of 42 kDa was detected as a major component in the 43 kDa band together with gamma actin, alpha skeletal muscle actin, and tubulin alpha-1B chain, where the coverage of GS by the tryptic peptides was 76.9% (see Supplementary Fig. S5A, B and Table S2). Detection of GS in these three distinct bands was probably caused by a mobility shift due to its modification by ubiquitination^17^. These data suggest that GS, a protein not homologous to Bcnt, was the most promising candidate for the 43 kDa protein recognized by the polyclonal anti-BCNT-C Ab. It should be noted that the anti-BCNT-C Ab with cross-reactivity to GS was generated in seven of eight guinea pigs immunized with the 18-mer BCNT-C peptide in two independent immunizations, making it unlikely that generation of the anti-BCNT-C Ab with such a cross-reactivity was accidental.

### Recognition of glutamine synthetase by the anti-BCNT-C Ab

**Figure 3:**
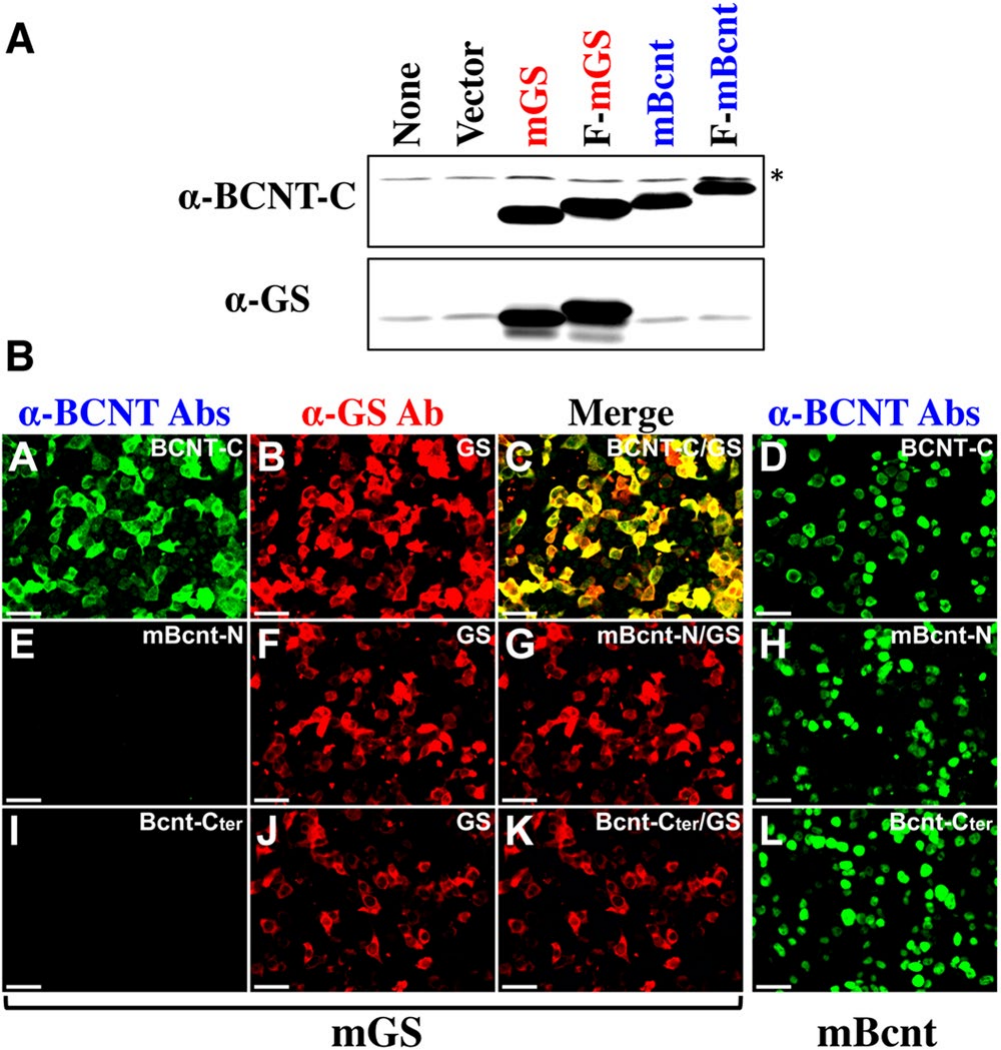
Specific recognition of GS by the anti-BCNT-C Ab. (**A**) HEK239T cells were transfected with plasmids encoding Flag-tagged or tag-free mouse GS (F-mGS, mGS), mouse Bcnt/Cfdp1 (F-mBcnt, mBcnt), respectively, and their extracts were subjected to Western blotting by double probing with the anti-BCNT-C Ab (upper panel) and the anti-GS Ab (lower panel). Both signals were detected by a fluorescent method. None and Vector indicate a vehicle and a blank vector control, respectively. **(B)** HEK293T cells were transfected with plasmids encoding mGS (A–C, E–G, I–K) or mBcnt (D,H,L) and immunostained with the anti-BCNT-C Ab (A,D), anti-mBcnt-N Ab (E,H), anti-Bcnt-Cter Ab (I,L), or anti-GS Ab (B,F,J). C, G, and K are merged figures of A with B, E with F, and I with J, respectively. Scale bars indicate 50 μm. The asterisk indicates non-specific band.

To examine whether the anti-BCNT-C Ab really cross-reacts with GS, we carried out Western blot analysis using two distinct GS sources: a purified recombinant His-tagged human GS expressed in *Escherichia coli* (*E*. *coli*) and extracts of skeletal muscle of FOXO1-transgenic mice, which preferentially increase the mRNA and protein expressions of GS^18^. These results demonstrate that the Ab detected the same size band at a comparable level as a canonical anti-GS Ab does (see Supplementary Fig. S6). Considering the effect of a His-tag added to a recombinant protein, we also expressed tag-free recombinant mouse GS and Bcnt or those with various tags, such as Flag, enhanced green fluorescent protein (EGFP), and mCherry, in HEK293T cells to examine multilaterally the cross-reactivity of the anti-BCNT-C Ab with GS, and then these recombinant proteins were analyzed by Western blotting and immunocytochemistry. Because the anti-BCNT-C Ab recognized GS regardless of type or presence or absence of tags, representative results of tag-free and Flag-tagged mGS and mBcnt are shown in Fig. 3A, which clearly show that the anti-BCNT-C Ab specifically recognized both Bcnt and GS. Additionally, we further examined by immunocytochemistry whether the cross-reactivity to GS *in situ* is specific for the anti-BCNT-C Ab using the anti-Bcnt-Cter Ab and the anti-mBcnt-N Ab. While the anti-BCNT-C Ab can specifically immunostain exogenously expressed GS in HEK cells, but not the two other anti-Bcnt Abs (Fig. 3B). The anti-BCNT-C Ab shows a strong immunoreactivity *in situ* as shown by positive immunostaining of Bergmann glia, a type of astrocyte, in the rat cerebellum (see Supplementary Fig. S7), which is consistent with previous reports that GS is a marker of astrocytes^19^. Other data are described in supporting information, which show that some characteristics of the immunoreactant recognized by the anti-BCNT-C Ab are also consistent with those previously reported for GS (see Supplementary Fig. S8, refs^20,21^). Because we confirmed that the anti-BCNT-C Ab really recognizes GS, a protein not homologous to Bcnt, we conducted epitope analysis of GS to investigate the mechanisms for such a peculiar cross-reactivity.

### Epitope analysis of GS for cross-reactivity of the anti-BCNT-C Ab

**Figure 4:**
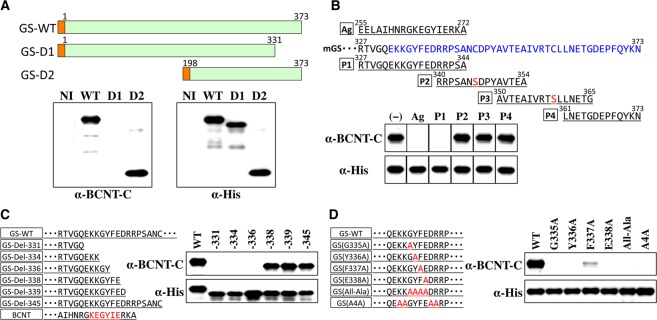
Core sequence of the epitope recognized by the anti-BCNT-C Ab on GS. (**A**) Upper panel: Schematic structures of full-length mGS (GS-WT) and its two deletion mutants (GS-D1 and GS-D2). Orange and light green boxes indicate His-tag and the amino acid residues of GS, respectively. Figures above each box represent the number of amino acids constituting the coding region. Lower panels: Western blotting of extracts of *E*. *coli* expressing GS-WT (WT), GS-D1 (D1), or GS-D2 (D2) and non-induced *E*. *coli* (NI) by the anti-BCNT-C Ab. The membrane was reprobed with anti-His-tag Ab to confirm the amounts of applied GS mutants. **(B)** Upper panel: Amino acid sequences of the antigen peptide (Ag) used to raise the anti-BCNT-C Ab, the C-terminal region of mouse GS and four peptide fragments (P1–P4) used for competition experiment. To avoid dimer formation, two cysteine residues of GS peptides were replaced with serine (red letters). Lower panel: Western blotting of extracts of *E*. *coli* expressing GS-WT with the anti-BCNT-C Ab preincubated with 5 μM of Ag, each GS peptide (P1-P4) or DMSO for negative control (−) as indicated on the top of each lane. An anti-His-tag Ab was used in the same way as in (A). **(C**,**D**) Western blotting of extracts of *E*. *coli* expressing GS-WT, six deletion GS mutants (C) or alanine substitution mutants of one or four amino acids (in red letters) (D) with the anti-BCNT-C or anti-His-tag Ab in the same way as in (A). Red letters in (C) indicate an amino acid sequence in antigen peptide for anti-BCNT-C Ab, which is similar to KKGYFE in P1 peptide of GS.

First, we prepared several deletion mutants of GS protein in *E*. *coli* to identify the region recognized by the anti-BCNT-C Ab on a GS molecule. Representative results of Western blotting are shown in Fig. 4A. While a clear positive reaction was detected for a GS mutant lacking almost half of the N-terminal amino acids (D2), no reaction was observed for a GS mutant lacking C-terminal amino acids 332–373 (D1), strongly suggesting that the epitope is localized in the C-terminal region of GS. To further narrow down the predicted epitope region, we employed competition experiments using four peptides with short overlapping amino acids (P1–P4 in Fig. 4B, upper panel, see Supplementary Fig. S9) derived from the C-terminal region of GS. Western blot analysis using the preincubated anti-BCNT-C Ab with each peptide or the antigen peptide showed that only P1 among the four peptides strongly inhibited the cross-reactivity of the Ab with GS similar to the antigen peptide (Fig. 4B, lower panel). These results strongly suggest that the epitope must be included in the P1 peptide sequence. Furthermore, to identify key amino acids involved in the cross-reaction, we prepared graded deletion mutants of GS and examined their reactivity with the anti-BCNT-C Ab by Western blotting. As shown in Fig. 4C, the cross-reactivity was clearly observed for the Del-338 mutant but was conspicuously absent for the Del-336 mutant. This result indicates that key amino acids for the cross-reaction are limited to RTVGQEKKGYFE in the P1 peptide sequence. We noticed that KKGYFE of GS is similar to KEGYIE of antigen peptide for anti-BCNT-C Ab, especially GYFE of GS and GYIE of BCNT (Fig. 4C, lower left), and we examined the cross-reactivity with a focus on GYFE of GS using alanine substitution mutants of GS. Whereas weak but significant reactivity was detected for the F337A mutant, no reactivity was detected for other alanine substitution mutants including E338A (Fig. 4D). These results clearly show that GYFE (hereinafter called “the core amino acids”) is required for the cross-reactivity of the anti-BCNT-C Ab with GS. As shown in Fig. 4D, mutant A4A also lacked reactivity with the Ab, suggesting the possibility that K333 and/or K334 may be included in the epitope.

### Evaluation of the affinity of the anti-BCNT-C Ab to GS and BCNT

**Figure 5:**
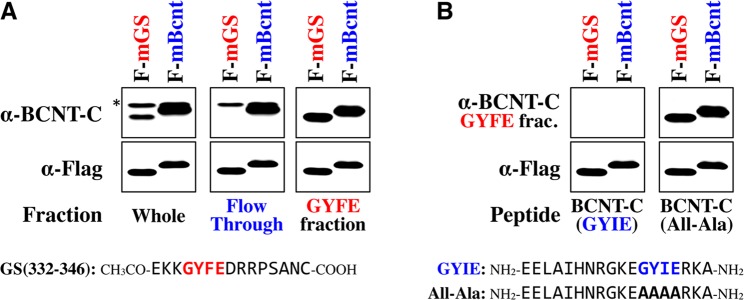
Isolation of the subpopulation of the polyclonal anti-BCNT-C Ab, which cross-reacts with GYFE of GS, and its affinity to GYFE of GS and GYIE of Bcnt. The subpopulation of the polyclonal anti-BCNT-C Ab cross-reacting with GYFE of GS was isolated by an affinity column using GS(332–346) peptide (see MATERIALS AND METHODS). Extracts of HEK293T cells expressing Flag-mGS (F-mGS) or Flag-mBcnt (F-mBcnt), which were adjusted to show an equal immunoreactivity to anti-Flag Ab, were subjected to Western blotting. The membrane was first reacted with anti-Flag Ab to confirm loading amounts (lower panels in *A* and *B*). After stripping, **(A)** the membranes were reprobed with untreated (Whole), flow-through (Flow Through) or affinity-purified (GYFE fraction) Abs from the polyclonal anti-BCNT Ab by GS(332–346) peptide affinity column. **(B)** The membranes were reporobed with the GYFE fraction pre-incubated using BCNT-C peptide (BCNT-C(GYIE)) or BCNT-C peptide replacing GYIE with Alanine (BCNT-C (All-Ala)). The asterisk indicates non-specific band.

Next, we evaluated the affinity of the anti-BCNT-C Ab to GS and BCNT by semi-quantitative Western blotting of Flag-tagged mouse GS (F-mGS) and Bcnt (F-mBcnt) normalized with an anti-Flag-tag Ab (Fig. 5). A subpopulation of the polyclonal anti-BCNT-C Ab, which cross-reacts with GS, was fractionated by a column of agarose resin cross-linked with GS(332–346) peptide including the core amino acids, GYFE (shown in Fig. 5A). The binding fraction (GYFE fraction) was isolated in about 20% yield as a protein weight, indicating that the subpopulation of the polyclonal anti-BCNT-C Ab cross-reacting with the core amino acids, GYFE, of GS occupied about one fifth. Consistent with these results, the normalized immunoreactivity of the anti-BCNT-C Ab for F-mGS is estimated to be approximately one fifth compared with that for F-mBcnt (Fig. 5A, left). Furthermore, the GYFE fraction from the anti-BCNT-C Ab reacted to F-mGS and F-mBcnt with equivalent immunoreactivity (Fig. 5A, right), whereas the flow-through fraction did not react to F-mGS (Fig. 5A, middle). These results suggest that the subpopulation of the polyclonal anti-BCNT-C Ab cross-reacting with GS, derives from only Ab molecules recognizing epitopes including the core amino acids, GYFE. On the other hand, the immunoreactivity of the GYFE fraction was inhibited with a peptide EELAIHNRGKEGYIERKA-NH_2_, BCNT-C (GYIE) in Fig. 5B, but not with its mutant replacing GYIE with Alanine, BCNT-C (All-Ala) in Fig. 5B. This result shows that the subpopulation of the polyclonal anti-BCNT-C Ab cross-reacting with GYFE of GS specifically reacts to GYIE of Bcnt. We also isolated the subpopulation from the anti-BCNT-C Ab by affinity purification on the membrane using full-length recombinant proteins of wild-type GS or All-Ala GS mutant expressed in *E*. *coli* (see Supplementary Fig. S10). These results indicate that a certain subpopulation exists in the polyclonal anti-BCNT-C Ab that reacts to both GS and Bcnt with similar affinities, and that GYFE of GS composes a core sequence of the epitope required for the cross-reactivity of the anti-BCNT-C Ab. It should be noted that the Ab recognized both GS and Bcnt with similar immunoreactivities in spite of the difference between Phe (GYFE) for GS and Ile (GYIE) for Bcnt.

### Spatial similarity of stereochemical environment between GS epitope and BCNT-C antigen

**Figure 6:**
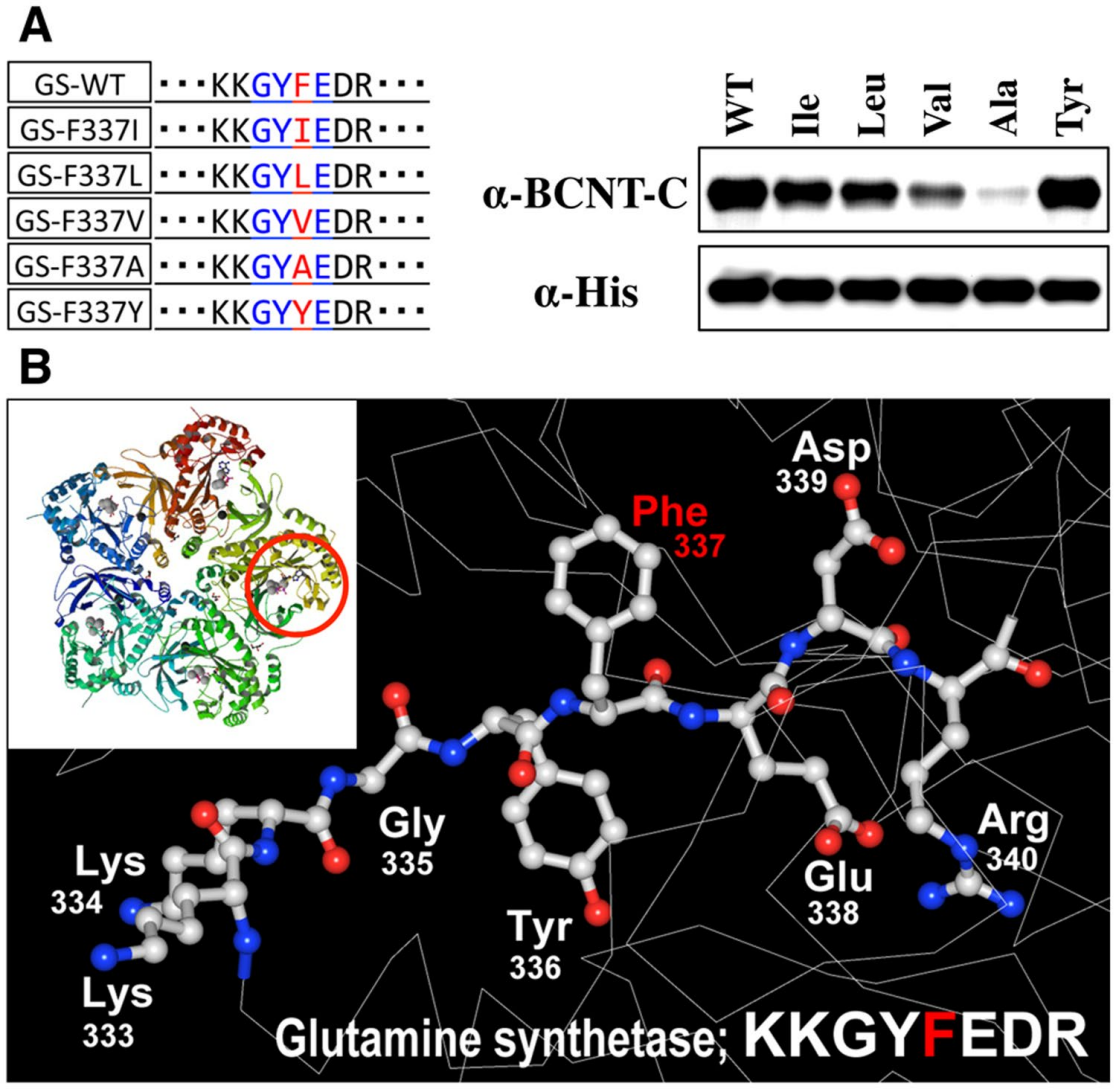
Role of Phe-337 in stereochemical environment of core amino acids of common epitope. **(A)** Western blotting was carried out as described in Fig. 4A using the extracts *of E*. *coli* expressing wild type GS (GS-WT) or its substitution mutants of F337 with various amino acids as shown in upper left panel and on the top of each lane. Blue letters indicate the core amino acids and red letters denote the respective substituted amino acids. **(B)** The inserted picture at the left top shows top-view of one pentameric ring for human GS consisted of two pentameric rings (PDB code 2OJW). The red circle depicts the location of the one of the core amino acids, GYFE, in the five identical subunits. 3D-layout of the KKGYFEDR peptide in GS displaying the spatial arrangement of the common epitope region. This layout was drawn using Waals (Altif Laboratories) based on a data of X-ray crystallography (PDB code 2OJW, ref.^22^) of human GS.

To understand the similar immunoreactivities of the anti-BCNT-C Ab against both GS and Bcnt in spite of the one amino acid difference of the core sequence, we considered the possibility that the cross-reactivity was caused by a spatial similarity of the stereochemical environment between the BCNT-C antigen peptide and a region of GS including GYFE. As described above, weak but significant immunoreactivity was detected for an alanine substitution mutant of GS (F337A) but not for other alanine-substitution mutants (Fig. 4D). Taken together, we conjectured that F337 of GS is not directly involved in the interface of the antigen-Ab interaction between GS and the anti-BCNT-C Ab. To investigate how F337 actually participates in the antigen-Ab interaction, we prepared substitution mutants of F337 of GS with various amino acids having different steric bulks (the amount of space that side chain atoms of amino acids occupy) or polarities and tried to semi-quantitatively analyze the effect of those substitutions on stereochemical and physiochemical changes of GS by Western blotting. As shown in Fig. 6A, when four hydrophobic and branched-chain amino acids were substituted in the order of steric bulk from isoleucine to alanine, the cross-reactivity of the anti-BCNT-C Ab was attenuated with an accompanying decrease in the steric bulk of the substituted amino acid, but it was not completely abolished. This result further supports our conjecture that F337 of GS is not directly engaged in the contact site of the antigen-Ab interaction but importantly constitutes the stereochemical environment of the epitope. Figure 6B shows a 3D layout of a region around GYFE of the common epitope of GS, which was drawn based on X-ray crystallography data of GS (PDB code 2OJW, ref.^22^). Note that Phe (F337) resides on the opposite side of neighboring Y336 and E338, occupying rather a space by its aromatic ring. This arrangement of F337 supports our speculation above that this amino acid is not directly involved in the contact site of the antigen-Ab interaction, but has a steric bulk effect on the stereochemical environment of the epitope. On the other hand, the cross-reactivity was approximately equal to the F337Y mutant as compared with the wild-type GS (F337) (Fig. 6A). These data suggest that the polarity of tyrosine, a hydroxyl group adduct of phenylalanine, does not significantly affect the stereochemical environment of the epitope. In conclusion, the results obtained strongly suggest that the spatial similarity of stereochemical environments formed by the core amino acids of common epitopes of GS and Bcnt is critically important in the cross-reaction of the anti-BCNT-C Ab with GS.

## Discussion

In this paper, we showed that the anti-BCNT-C Ab quite specifically cross-reacted with GS, which is a protein not homologous to Bcnt, in addition to the original target molecule. GS is present predominantly in the brain where it participates in the metabolic regulation of glutamate^14,23^. The anti-BCNT-C Ab recognized exogenous GS expressed in HEK cells and also showed strong immunoreactivity in astrocyte of adult rat cerebellum (see Supplementary Fig. S7), consistent with the localization of GS in brain previously reported^20^. It is most likely that strong immunoreactivities of the anti-BCNT-C Ab in Leydig cells of bovine testis^24^ and astrocytes of rat brain, we had conjectured as Bcnt/Cfdp1 so far, are immunoreactivities for endogeneous GS^20,25^. In addition to its strong immunoreactivity, this Ab detects GS under different denaturing conditions: thermally and reductively denatured GS in Western blotting, chemically fixed GS *in situ* in immunocytochemistry and immunohistochemistry, and a more naïve form of GS in aqueous solution in immunoprecipitation. These data strongly suggest that the epitope including GYFE, which is reactive to the anti-BCNT-C Ab, localizes on the surface of the native GS.

Although no significant homology of amino acid sequences was found between Bcnt and GS, we were able to identify the core amino acid sequence, GYFE, localized in a limited region of the C-terminal of GS, that is necessary for the cross-reactivity of the anti-BCNT-C Ab (Fig. 4C). We conjectured that this sequence has an epitope in common with GYIE in the immunogen peptide for the anti-BCNT-C Ab. This conjecture implies that replacement of an aromatic amino acid, Phe, of GYFE with a branched chain amino acid, Ile, maintains the epitope environment. This assumption is consistent with the results shown in Fig. 6A. GS mutants with a substitution of Phe with Ile, Leu, or Tyr showed comparable cross-reactivity of the anti-BCNT-C Ab, whereas replacement of Phe with a smaller amino acid, Val or Ala, resulted in less reactivity with the Ab, indicating that the steric bulk of each amino acid is critical for the reactivity of the epitope with the Ab. Moreover, the result also indicates that the steric bulk of the aromatic ring of F337 or Tyr of the F337Y mutant of GS, but not the polarity of amino acids, is important to maintain the stereochemical environment for the cross-reactivity of the anti-BCNT-C Ab with GS. Therefore, this result strongly suggests that Phe in GYFE (GS) and Ile in GYIE (Bcnt) play a role in maintaining the spatial arrangement of the common epitope but are not directly involved in the contact site of the antigen-Ab interaction. This speculation is consistent with a previous report that F337 resides on the opposite side of neighboring Y336 and E338^22^, and that each is involved in binding ATP and glutamate, a substrate of GS, respectively, in the active site of the GS complex (Fig. 6B). It is also reported that though F337 in the active site of GS is highly conserved among species, it is replaced by Leu in *Mycobacterium tuberculosis* and Ile in *Salmonella typhimurium*^13,22^, suggesting that both the function and stereochemical environment of the active site of GS are maintained even if F337 is replaced with Leu or Ile. These data lead to the speculation that the core amino acids of the common epitopes, GYFE located in this site of GS and GYIE of Bcnt, constitute similar stereochemical environments.

It is also noted that the conjectured core amino acids of the common epitope are bracketed by charged amino acid residues such as lysine and arginine (Fig. 4C, lower left). The hydrophilicity of these charged residues may bring the core amino acids of each epitope to the surface of GS or BCNT. Elucidation of this characteristic of epitopes awaits further studies.

It is well known now that whereas cross-reactions of Abs with structurally unrelated peptides of off-targets frequently occur in binding assays of Abs using small peptides, such cross-reactions rarely occur in those using protein fragments and in Western blot analysis^9^. It has been generally believed that Abs recognize linear epitopes of target molecules under the denatured condition of SDS-PAGE, but some Abs can also react to proteins via conformational epitopes, probably depending on the protein renaturation on transferred membranes after denaturation in SDS-PAGE^26,27^. The anti-BCNT-C Ab reacted to each GS mutant for Phe of GYFE in a steric bulk-dependent manner (Fig. 6A), in contrast to the loss of immunoreactivities in alanine-substituted mutants for Gly, Tyr, and Glu of GYFE (Fig. 4D). These results reflect differences in whether these amino acids exist at the contact site for the Ab (Gly, Tyr, and Glu) or not (Phe). This result, which is consistent with the configuration of the core amino acids displayed in the 3D structure of GS (Fig. 6B), suggests that Bcnt and GS are partially renatured and that their conformations are partially maintained on transferred membranes in Western blot analysis. Such renaturation of Bcnt and GS is inferred to be one of the reasons why we were able to elucidate the similarity of stereochemical environments between GYIE of Bcnt and GYFE of GS by Western blot analysis.

Recent reports including this paper indicate that cross-reactions of Abs with off-target molecules cannot be avoided, even when choosing a very specific amino acid sequence as an immunogen^9,28^. Therefore, we have to pay attention to the fact that Abs do not necessarily recognize only “on-targets” but potentially react with “off-targets” that comprise similar stereochemical or physicochemical environments as that of on-target molecules. Recent advances in computational and bioinformatic analyses using extensively accumulated data of epitope-paratope interfaces make it possible to group T cell receptors of common specificity using grouping algorithms of lymphocyte interactions by paratope hotspots^29^. Furthermore, these advances have also revealed the functional redundancy of epitopes based on physicochemical similarities at a level of amino acid residues involved in the antigenic cross-reaction^30^. In addition to these accumulated data, further comprehensive understanding of the physicochemical environment of epitope-paratope interfaces including both “on-targets” and “off-targets” may make it possible to evaluate and predict more accurately antigenicity and immunogenicity, and these advances will bring about therapeutic Abs and vaccines with high specificity and without adverse effects.

## Materials and Methods

### Ethical approval

All the genetic recombination experiments and all the animal experiments in the present study were approved by the Genetic Recombination Experiment Safety Committee and the Animal Care and Use Committee, respectively, of Tokushima Bunri University.

### Reagents

Generation of anti-BCNT-C Ab, anti-mBcnt-N Ab, and anti-Bcnt-Cter Ab is described in Supplementary Materials and Methods. Abs against Flag tag (DYKDDDDK tag; 014-22383) and GS (GTX109121) were obtained from Wako Pure Chemical Industries and GeneTex, respectively. Unstained or prestained SDS-PAGE molecular weight standard markers (Bio-Rad) were used differently for each experiment.

### Construction of expression vectors for mGS and mBcnt

A plasmid carrying Myc-tagged mGS was a gift from Dr. T. Araki (Natl. Inst. Neurosci., Tokyo)^17^. Using cloning primers (see Supplementary Table S3-1), mGS and mBcnt cDNAs were amplified from cDNA, which was reverse transcribed from the total RNA of the brain of an adult male C57BL/6 J mouse using dT-primer and inserted into mammalian expression vectors, as described in Supplementary Materials and Methods. All the expression vectors for GS mutants were constructed and used as described in Supplementary Materials and Methods. Among GS mutants, F337A and an all-Ala replacement mutants of 355–358 (GYFE) were confirmed by MALDI-TOF MS and LC-MS/MS after digestion with Asp-N protease or trypsin.

### Cell culture and transfection

HEK-293T cells were maintained at 37 °C, 5% CO_2_, in high glucose (4.5 g D-glucose/L) DMEM-GlutaMAX-I (Thermo Fisher Scientific) supplemented with 10% Fetal bovine serum and 50 mg/L gentamicin. HEK-293T cells were plated in 6-well plates at ~60% confluency and transfected with an expression vector DNA (0.5 μg/well) using PLUS reagent (0.5 μL/well) and Lipofectamine LTX reagent (1.2 μL/well) (Thermo Fisher Scientific).

### Preparation of extracts of *E*. *coli* expressing GS and its mutants

Culture of *E*. *coli* and induction of coded molecules by cDNAs in pCold II vector were described previously^11^. *E*. *coli* were harvested and washed with PBS by centrifugation at 5,000 × g for 5 min. After addition of 50 μL H_2_O per pellet derived from 1 mL culture, resuspended cells were mixed with an equal volume of 2-fold concentrated lysis buffer (40 mM Tris-HCl buffer (pH 7.6) containing 2% SDS) and immediately boiled for 3 min. Extracts were sonicated by a Bioruptor (BM Equipment) in an ice-water bath (15 × 10 s pulses at 10 s intervals) and followed by centrifugation at 28,000 × *g* for 10 min. Supernatants were subjected to Western blot analysis.

### Immunoblotting

Procedures of SDS-PAGE and blotting onto membranes were essentially the same as previously described^11^ except that a low fluorescence membrane (Immobilon-FL, Merck) was used in the fluorescent method. Immunodetections were carried out by chemiluminescent, colorimetric, and fluorescent methods using horseradish peroxidase (HRP)-, alkaline phosphatase (AP)- and fluorescent dye-conjugated secondary Abs, respectively. The secondary IgGs used are listed in Supplementary Materials and Methods. Tris-buffered saline supplemented with 0.1% Tween 20 (TBT) was used as a wash buffer in all three methods. In the chemiluminescent method, immunoreaction was carried out in TBT containing 5% or 10% skim milk and the immunoreactivity was detected by a scanner (GeneGenome, Syngene Bio Imaging) using Immobilon (Merck), ImmunoSTAR Zeta, or LD (Wako Chem.) as a substrate. In the colorimetric method, immunoreaction was carried out in TBT containing 0.2% casein (I-Block, Thermo Fisher Scientific), and the immunoreactivity was detected by an LAS-3000 Imager (Fuji-Film) using 5-bromo-4-chloro-3-indolyl phosphate/nitro blue tetrazolium (Sigma) as a chromogenic substrate. In the fluorescent method, the immunoreaction was carried out in TBT containing 0.2% casein, and the immunoreactivity was detected by an Odyssey 700 CLX Infrared Imaging system (LI-COR Biosciences). Membranes of bound Abs for reprobing were stripped by shaking membranes in 62.5 mM Tris-HCl (pH 6.8), 2% SDS, and 100 mM 2-mercaptoethanol for 1 h at 70 °C. To avoid inhibition by former enzymatic reaction products, reprobing was carried out only after detection by the fluorescent method.

### Isolation of a subpopulation of the polyclonal anti-BCNT-C Ab, which cross-reacts with GYFE of GS

GS(332–346) peptide, acetylated-EKKGYFEDRRPSANC-COOH (0.75 mg, 91.2% purity, obtained from AnyGen), was coupled to 1 mL of SulfoLink Coupling Resin (Thermo Fisher Scientific) packed in a column (MoBiTec) according to a manufacture’s protocol. The anti-BCNT-C Ab of 4.45 mg diluted in 10 mL with Binding/Wash buffer (20 mM PBS (pH 7.0) containing 0.05% sodium azide) passed through the column 5 times and a flow-through fraction was obtained. After washing the resin with 50 mL of Binding/Wash buffer, the bound fraction (GYFE fraction) was eluted with 6 mL of 100 mM Glycine (pH 3.0) and immediately neutralized with Neutralization buffer (1 M Tris-HCl (pH 8.5), 1.5 M NaCl, 5 mM EDTA). The protein concentrations of the binding fraction (GYFE fraction) and the flow-through fraction were determined by Bicinchoninic acid (BCA) assay kit (Thermo Fisher Scientific) using bovine serum albumin as a standard after buffer exchange and concentration by ultrafiltration using Amicon Ultra-4, 30 kDa (Merck Millipore). Their protein concentrations were 0.78 mg and 2.80 mg, respectively, with total recovery of 80.7%.

### Competitive experiments for evaluation of the specificity of the GYFE fraction to GYIE of Bcnt

Extracts of HEK293T cells expressing Flag-mGS (F-mGS) or Flag-mBcnt (F-mBcnt) were adjusted to show an equal immunoreactivity to anti-Flag Ab, then subjected to Western blotting. The membrane was first reacted with anti-Flag Ab to confirm loading amounts. After stripping, the membranes were reporobed with the GYFE fraction (1.33 nM as a IgG) pre-incubated with BCNT-C peptide (NH_2_-EELAIHNRGKEGYIERKA-NH_2_, 92.3% purity, from AnyGen) or BCNT-C peptide replacing GYIE with Alanine (NH_2_- EELAIHNRGKEAAAARKA-NH_2_, 98.3% purity, from AnyGen) at a final concentration of 25 μM.

### Mass spectroscopy analysis

The CBB stained protein bands in the gel were excised and de-stained. The protein bands were digested with trypsin (TPCK-treated, Worthington Biochemical) at 37 °C for 12 h in 50 mM Tris-HCl (pH 8.0). The digests were analyzed by nano LC–MS/MS using a Q Exactive mass spectrometer exactly as described previously^11^.

### Immunocytochemistry

Transfected HEK-293T cells (see “*Cell culture and transfection”*) were plated on glass slides (Millicell EZ 4-well glass slides, Merck Millipore) coated with poly-D-lysine (Sigma-Aldrich). After 30 h, the cultured cells were fixed with ice-cold 4% paraformaldehyde (PFA) in 0.1 M phosphate-buffer (PB, pH 7.2) for 15 min and subjected to double immunostaining as described in the “*Immunohistochemistry”* section of Supplementary Materials and Methods. First, cells were stained with guinea pig anti-BCNT-C Ab (1:500) and visualized by FITC-conjugated donkey anti-guinea pig IgG (1:50, Jackson ImmunoResearch). The second staining was done with rabbit anti-GS Ab (1:1000, GeneTex) visualized by Cy3-conjugated donkey anti-rabbit IgG (1:200, Jackson ImmunoResearch). Immunostained cells were examined by confocal laser scanning microscopy (FV1000, Olympus).

## Supplementary information

Supplementary Information
