# Author Correction: A spatial similarity of stereochemical environments formed by amino acid residues defines a common epitope of two non-homologous proteins

**DOI:** 10.1038/s41598-020-68279-6

**Published:** 2020-07-01

**Authors:** Kentaro Nakashima, Shintaro Iwashita, Takehiro Suzuki, Chieko Kato, Toshiyuki Kohno, Yasutomi Kamei, Motoki Sasaki, Osamu Urayama, Yoshiko Ohno-Iwashita, Naoshi Dohmae, Si-Young Song

**Affiliations:** 1 Institute of Neuroscience, Tokushima Bunri University, Kagawa, 769-2193 Japan; 2 Department of Pharmacy, Iwaki Meisei University, Fukushima, 970-8551 Japan; 3 Biomolecular Characterization Unit, RIKEN Center for Sustainable Resource Science, Saitama, 351-0198 Japan; 4 Department of Biochemistry, Kitasato University School of Medicine, Kanagawa, 242-0374 Japan; 5 Laboratory of Molecular Nutrition, Graduate School of Environmental and Life Science, Kyoto Prefectural University, Kyoto, 606-0823 Japan; 6 Department of Basic Veterinary Medicine, Obihiro University of Agriculture and Veterinary Medicine, Hokkaido, 080-8555 Japan; 7 Faculty of Health Sciences, Tsukuba International University, Ibaraki, 300-0051 Japan

Correction to:* Scientific Reports* 10.1038/s41598-019-51350-2, published online 15 October 2019

This Article contains errors in the Supplementary Information file.

In the Materials and Methods section, a subsection entitled “Construction of expression vectors for mGS and mBcnt”, which should be placed between the “Generation of the anti-BCNT-C Ab, anti-Bcnt-Cter Ab and anti-mBcnt-N Ab” and “Construction of expression vectors for deletion and substitution mutants of mGS” subsections was omitted and appears below:

**Construction of expression vectors for mGS and mBcnt**

cDNAs were synthesized from total RNA of the whole brain of an adult male C57BL/6J mouse by using SuperScript III reverse transcriptase (Thermo Fisher Scientific) and oligo(dT) according to the manufacture’s protocol. mGS cDNA and mBcnt cDNAs were respectively amplified from the mouse brain cDNA using KAPA HiFi HotStart DNA polymerase (KAPA Biosystems) and their specific cloning primers (see Supplementary Table S3-1) under the following conditions; initial denaturation for 3 min at 95 °C and 40 cycles of denaturation for 20 sec at 98 °C, annealing for 20 sec at 65 °C and extension for 1 min at 72 °C. PCR products were inserted into following four kinds of mammalian expression vectors by using restriction enzymes *Bgl*II or *Bam*HI for 3′ end and *Xho*I for 5′ end: BsrGI-MCS-pcDNA3.1 (Accession No. LC311017), Flag-MCS-pcDNA3.1 (Accession No. LC311018), Flag-EGFP-MCS-pcDNA3.1 (Accession No. LC311019) and Flag-mCherry-MCS-pcDNA3.1 (Accession No. LC311020). ORF sequences of all constructs were confirmed by BigDye Terminator V3.1 Cycle Sequencing Kit (Thermo Fisher Scientific) using the sequencing primers (Table S3-3).

Additionally, in the section entitled “Construction of expression vectors for deletion and substitution mutants of mGS”, the sentence “PCR products were also inserted into one of the following three kinds of mammalian expression vectors by using restriction enzymes *Bgl*II or *Bam*HI for 3′ end and *Xho*I for 5′ end: BsrGI-MCS-pcDNA3.1 (Accession No. LC311017), Flag-MCS-pcDNA3.1 (Accession No. LC311018) and Flag-mCherry-MCS-pcDNA3.1 (Accession No. LC311020).” should have been omitted.

In the legend for Table S1, entitled “Table S1: Properties of four types of anti-Bcnt Abs” the sentence,

“As for details of preparation of Abs, see Supplementary Materials and Methods online.”

should read:

“As for details of preparation of Abs, see Supplementary Materials and Methods.”

Additionally, in the figure legend for Figure S8,

“Cellular components of adult rat whole cerebrum were fractionated using a subcellular fractionation kit (Invitrogen).”

should read:

“Cellular components of adult rat whole cerebrum were fractionated using a subcellular fractionation kit (ProteoExtract Subcellular Proteome Extraction Kit, Merck Millipore).”

In Table S3-3, four primers were omitted. The correct Supplementary File is appended to this notice.

## Supplementary information

Supplementary information

