# Supplementary material for: A spatial similarity of stereochemical environments formed by amino acid residues defines a common epitope of two non-homologous proteins
Source: Sci Rep. 2019 Oct 15;9:14818. doi: 10.1038/s41598-019-51350-2 (PMC6794283; doi:10.1038/s41598-019-51350-2)

SUPPLEMENTARY INFORMATION:

TITLE:

A spatial similarity of stereochemical environments formed by amino acid residues defines a common epitope of two non-homologous proteins

AUTHORS:

Kentaro Nakashima[1*], Shintaro Iwashita[1*, 2], Takehiro Suzuki[3], Chieko Kato[1], Toshiyuki Kohno[4], Yasutomi Kamei[5], Motoki Sasaki[6], Osamu Urayama[7], Yoshiko Ohno-Iwashita[2], Naoshi Dohmae[3] and Si-Young Song[1*]

AFFILIATIONS:

1. Institute of Neuroscience, Tokushima Bunri University, Kagawa 769-2193, Japan
2. Department of Pharmacy, Iwaki Meisei University, Fukushima 970-8551, Japan
3. Biomolecular Characterization Unit, RIKEN Center for Sustainable Resource Science, Saitama 351-0198, Japan
4. Department of Biochemistry, Kitasato University School of Medicine, Kanagawa 242-0374, Japan
5. Laboratory of Molecular Nutrition, Graduate School of Environmental and Life Science, Kyoto Prefectural University, Kyoto 606-0823, Japan
6. Department of Basic Veterinary Medicine, Obihiro University of Agriculture and Veterinary Medicine, Hokkaido 080-8555, Japan
7. Faculty of Health Sciences, Tsukuba International University, Ibaraki 300-0051, Japan

*** CORRESPONDING AUTHORS:**

Kentaro Nakashima (sections of molecular biology and biochemistry)

E-mail: nkentaro@kph.bunri-u.ac.jp

Phone: (81) 87-899-7484

Fax: (81) 87-894-0181

Shintaro Iwashita (section of biochemistry)

E-mail: siwast@kph.bunri-u.ac.jp

Phone: (81) 87-899-7484

Fax: (81) 87-894-0181

Si-Young Song (section of immunohistochemistry)

E-mail: sysong@kph.bunri-u.ac.jp

Phone: (81) 87-899-7484

Fax: (81) 87-894-0181

Institute of Neuroscience,

Tokushima Bunri University

1314-1 Sido, Sanuki, Kagawa, 769-2193, Japan

SUPPLEMENTARY MATERIALS AND METHODS

Instruments

We used SpectraMax M5TB and Agilent 2100 Bioanalyzer for protein and RNA quantification, ABI PRISM 3130/3130xl Genetic Analyzer for sequencing analyses and Odessey and LAS-3000 for digital imaging of fluorescent or chemiluminescent immunoblotting at the Center for Instrumental Analysis of Tokushima Bunri University.

Reagents and Antibodies

Recombinant human glutamine synthetase protein fused with 20 amino acids His-tag at N-terminus (enz-544, ProSpec),

HRP-conjugated anti-Guinea pig IgG (706-035-148, Jackson ImmunoResearch Lab), HRP-conjugated anti-Mouse IgG (715-035-151, Jackson ImmunoResearch Lab), HRP-conjugated anti-Rabbit IgG Ab (711-036-152, Jackson ImmunoResearch Lab), IRDye700™DX-conjugated anti-Guinea pig IgG (606-130-129, Rockland Immunochemicals), CF680-conjugated anti-Guinea pig IgG (20241, Biotium), CF770-conjugated anti-Rabbit IgG (20078, Biotium), CF770-conjugated anti-Mouse IgG (20077, Biotium)

Generation of the anti-BCNT-C Ab, anti-Bcnt-Cter Ab and anti-mBcnt-N Ab

To generate the anti-BCNT-C Ab, a peptide, EELAIHNRGKEGYIERKA, which corresponds to 18 amino acid residues (259-276) located at the C-terminal region of human BCNT, was used as an antigen. The antigen peptide, which has an additional cysteine in the N-terminus and a COOH group in the C-terminus, was synthesized with more than 93 %

purity. Two and six Hartley guinea pigs (5-6 week-old males) were immunized at different times by subcutaneous injection of 0.2 and 0.25 mg antigen peptide, respectively, which was coupled to keyhole limpet hemocyanin through the cysteine residue, supplemented with Freund's complete adjuvant. Typically, two booster injections with Freund's incomplete adjuvant were carried out on days 14 and 28. Whole blood was collected from each animal on day 38-52 and sera were prepared. All these processes were outsourced to Takara Bio. Two other anti-Bcnt/Cfdp1 Abs, designated anti-Bcnt-Cter Ab and anti-mBcnt-N Ab, were similarly generated in each three guinea pigs using a peptide of 10 amino acids, RDLRLSKMKP-COOH, located at the C-terminus of BCNT with an additional cysteine at the N-terminus and a peptide of 16 amino acids, acetyl-GEEQAEKTKGKRRKAQ, located at the N-terminal region of mouse Bcnt with an additional cysteine at the C-terminus, respectively. These peptides were synthesized by AnyGen. Immunization of animals and collecting whole blood were also outsourced to Takara Bio. The anti-BCNT-C Ab was affinity-purified from each serum of guinea pigs using a HiTrap NHS-activated HP column (GE Healthcare) coupled with the antigen peptide. The anti-Bcnt-Cter Abs and the anti-mBcnt-N Abs were purified from each serum by using SulfoLink Coupling Resine (Thermo Fisher Scientific) coupled with each antigen peptide through the additional cysteine.

Construction of expression vectors for deletion and substitution mutants of mGS

Expression vectors for deletion and substitution mutants of mGS were constructed from pCold II DNA plasmid carrying mGS according to a protocol of PrimeSTAR mutagenesis basal kit (Takara Bio) using PrimeSTAR GXL DNA polymerase or KAPA HiFi HotStart

DNA polymerase (KAPA Biosystems). PCRs were performed using 0.3 μM of each set of mutagenesis primers (Table S3-2) under either of following two conditions: 1) initial denaturation for 3 min at 95°C and 35 cycles of denaturation for 20 sec at 98 °C, annealing and extension for 3 min at 72°C when using KAPA HiFi HotStart DNA polymerase for a A4(AAGYFEAA) mutant, or 2) initial denaturation for 3 min at 95°C and 35 cycles of denaturation for 10 sec at 98°C, annealing for 15 sec at 60°C, and extension for 2 min at 68°C when using PrimeSTAR GXL DNA polymerase for other mutants. These PCR products were used for transformation of *E. coli* XL1-Blue after confirmation of their amplicon size by 0.8% TAE-agarose gel electrophoresis and purification by Wizard SV Gel and PCR Clean-Up System (Promega). All constructs obtained were expressed in BL21 (DE3) cells (Delphi Genetics S.A.) after confirmation of their ORF sequences and the promoter region using the designed primers (Table S3-3) and BigDye Terminator V3.1 Cycle Sequencing Kit (Thermo Fisher Scientific). PCR products were also inserted into one of the following three kinds of mammalian expression vectors by using restriction enzymes *Bgl*II or *Bam*HI for 3’ end and *Xho*I for 5’ end: BsrGI-MCS-pcDNA3.1 (Accession No. LC311017), Flag-MCS-pcDNA3.1 (Accession No. LC311018) and Flag-mCherry-MCS-pcDNA3.1 (Accession No. LC311020).

Preparation of an anti-BCNT-C peptide Ab-linked agarose

Anti-BCNT-C Ab-affinity agarose was prepared according to a protocol of Abcam (www.abcam.com/technical) by mixing 400 μg anti-BCNT-C Ab with 250 μL agarose (packed beads of Protein A/G PLUS-Agarose, Santa Crutz Biotech) and by coupling with dimethyl pimelimidate (Sigma-Aldrich). For preparation of a control agarose, the affinity

agarose resin described above was boiled in Hepes buffer (HB; Hepes-NaOH pH 7.5) for 5 min, and an unrelated calmoldulin-agarose (Sigma-Aldrich) was also used.

Preparation of protein extracts of cow, mouse and rat organs

Dissected pieces of a bovine brain (2-year-old female) and mouse brains (mixtures of 6 months to 1 year old C57Bl/6J mice) were frozen in liquid N_2 and stocked at -80°C until use. A piece of frozen tissues was crushed in a metal cylinder containing liquid N_2, and the resulted fine grains were homogenized in Hepes buffered saline (HBS; 10 mM Hepes-NaOH (pH 7.5), 150 mM NaCl) supplemented with protease inhibitor cocktail (Nacalai Tesque) and phosphatase inhibitors (10 mM NaF, 1 mM sodium vanadate, 12.5 mM β-glycerophosphate) with 40 strokes using a Dounce homogenizer. The crude extracts were centrifuged at 20,000 x *g* for 15 min at 4°C (Hitachi PA50, rotor AG-6512C) and the supernatant (S-20) was collected. Extracts of rat olfactory bulb [1] and skeletal muscle of FOXO1-transgenic mice and wild littermates [2] were prepared as previously reported.

Immunoprecipitation of the 43 kDa protein with the anti-BCNT-C Ab from bovine brain extracts in RIPA buffer or those boiled once in SDS buffer

Bovine brain S-100 extract [3] was mixed with an equal volume of two-fold concentrated RIPA buffer (final concentration: 10 mM Hepes-NaOH (pH 7.4), 150 mM NaCl, 1% sodium deoxycholate, 1% Triton X-100, 0.1% SDS, 5 mM Ethylenediaminetetraacetic acid (EDTA), 2.5 mM Ethyleneglycoltetraacetic acid (EGTA)) supplemented with inhibitors of proteinases and phosphatases, and the mixture was sonicated by a Bioruptor in an ice-water bath (15 x 10 s pulses at 10 s intervals), followed by centrifugation at 28,000 x *g* for 10 min.

Twenty μL *Staphylococcus aureus* suspension (IgG-Sorb, The Enzyme Center, binding capacity of 1.9 mg IgG per mL of reconstructed solution) was added to 1 mL extract, and the mixture was incubated for 30 min using a rotary shaker and centrifuged at 12,000 x *g* for 30 min. The supernatant was divided into two samples and each sample was incubated with an anti-BCNT-C Ab-linked agarose (10 μL packed volume) or a control agarose using a rotary shaker for 2 h and centrifuged. The supernatant was saved as the unbound fraction, and pellets were washed three times with RIPA buffer. Finally bound proteins were eluted from the agarose twice, each with 20 μL of 100 μM antigen peptide in the HB used for anti-BCNT-C Ab generation. On the other hand, the extract was resolved in 120 μL HB containing 1 mM 2-mercaptoethanol, 1 mM EDTA and 1% SDS (w/v, final concentration) and boiled for 4 min. Then Nonidet P-40 (hereinafter called "NP40") was added to the solution followed by adding HBS making a final concentration of both NP40 and SDS to 0.1%, respectively [4]. After treatment with IgG-Sorb, immunoprecipitation was carried out exactly the same as in RIPA buffer.

Isolation of the 43 kDa protein from bovine brain extracts

S-20 extracts of bovine brain (2.4 mg protein in 1 mL S-20) were fractionated by 20–55% ammonium sulfate (AS) precipitation at 4°C, and pellets were resuspended in 10 mM HB (pH 7.5) containing 0.5 mL of 1 M AS. The preparation was applied to a column packed with 1 mL phenyl-Sepharose CL-4B (GE Healthcare Life Science) by gravity flow. After washing the resin with 5 mL of 1 M AS, bound proteins were eluted with HB containing 2% n-octyl-β-D-glucoside (Dojindo Laboratories) and the eluate was collected (1 mL per tube) by gravity flow. The third to fifth fractions were subjected to acetone precipitation, and

pellets were washed three times with a mixture of acetone:H_2O = 4:1 and dried by a vacuum concentrator (Speedvac Concentrator, Savant) for 5 min. Obtained pellets were resolved in 120 μl HB containing 1% SDS (w/v), 1 mM 2-mercaptoethanol and 1 mM EDTA, boiled and subjected to immunoprecipitation as described above. The protein-bound agarose were washed three times with HBS containing 0.1% NP40 twice with HB, then eluted with HB containing 10 μM antigen peptides (50 μL). Elution was repeated three times and the eluates were pooled and subjected to acetone precipitation followed by SDS-PAGE.

Immunoprecipitation of the 43 kDa protein from extracts of rat olfactory bulb

Extracts of rat olfactory bulb (see, above) stored in 0.25 M sucrose at -80°C were thawed and concentrated by 60% AS precipitation to remove sucrose and resolved once in HBS, and then subjected to acetone precipitation. Boiling of the extracts in SDS, dilution with HBS containing 0.1% NP40, and immunoprecipitation were exactly the same as described in immunoprecipitation from bovine brain extracts.

Competitive experiment with peptides in Western blot analysis

Extracts of *E. coli* expressing His-tagged GS were subjected to Western blot analysis (100 ng/lane). The anti-BCNT-C Ab (2 nM) was preincubated with 5 μM antigen peptide or each GS peptide (P1–P4 described in Fig. 4B, which were synthesized at JPT Peptide Technologies and dissolved in DMSO) in blocking reagent (0.2% casein in TBT) at room temperature for 1 h. Western blotting was carried out using each preincubated Ab.

Immunohistochemistry

Rats were anesthetized by diethyl ether on postnatal day 56, subjected to transcardial pre-perfusion with saline warmed to 37°C containing 50 units/mL heparin sodium (Novo Nordisk) to inhibit blood coagulation, and systemically fixed by perfusion with ice-cold 4% paraformaldehyde (PFA) in 0.1 M phosphate buffer (PB, pH 7.2). Fixed brains were isolated, dissected, and post-fixed in the same fixative overnight. After being washed three times in 0.1 M PB, brains were immersed in 20% sucrose in 0.1 M PB at 4°C overnight for cryoprotection, embedded in O.C.T. compound (Sakura Finetechnical), and frozen by cooling with a mixture of dry ice and acetone. Frozen sections were cut at 8 μm and thaw-mounted on silane-coated glass slides. Sections were immunostained by an indirect double immunofluorescence method using guinea pig anti-BCNT-C Ab (1:100) in combination with rabbit anti-GS Ab (1:400, GeneTex), anti-GFAP Ab (1:400, DAKO) as an astrocyte marker or rabbit anti-Spot 35 Ab (1:400), a kind gift from Dr. T. Yamakuni (Tohoku University) [5], as a neuronal marker. All the blocking serum, antibodies, and nuclear staining dye were used at the indicated dilutions with 0.1 M PBS containing 0.2% Triton X-100. Sections were blocked with 5% normal donkey serum (Jackson ImmunoResearch) for 30 min at room temperature, then incubated with the anti-BCNT-C Ab at 4°C overnight. Anti-BCNT-C Ab immunoreactivity was visualized using FITC-conjugated or Alexa488-conjugated donkey anti-guinea pig IgG (Jackson Immuno Research). Then sections were incubated with rabbit anti-GS Ab, with rabbit anti-Spot 35 antibody or with anti-GFAP antibody similarly as described above. The immunoreactivity of these two antibodies was visualized using Cy3-conjugated donkey anti-rabbit IgG (Jackson ImmunoResearch). Finally, TO-PRO-3 (1:500, Molecular Probes) was used for

nuclear staining. Immunostained sections were examined by a confocal laser scanning microscopy (Olympus FV1000).

Isolation of an anti-BCNT-C Ab subpopulation specific to GS

Antibody molecules that specifically react with GS were isolated from the anti-BCNT-C Ab essentially according to a previously reported method [6]. In brief, extracts of *E. coli* expressing wild-type GS or All-Ala GS mutant were subjected to Western blotting (each 20 μg per lane). The immobilized membrane was cut into two strips (1 cm x 1.5 cm each) containing proteins with a molecular mass ranging between 37 and 50 kDa in each lane. After blocking with 5% skim milk in TBT for 1 h at room temperature and further for 30 min at 4°C, the strips were incubated with the anti-BCNT-C Ab (2.5 μg per 1.25 mL blocking solution) overnight at 4°C. After washing three times with TBT, bound Abs were recovered from membranes by overlaying chilled 100 mM glycine-HCl buffer (pH 2.5, 50 μL) on the membrane at 4°C for 2 min and immediately neutralizing with 2 M Tris (1.5 μL/50 μL). This procedure was repeated three times, and the collected solution was adjusted to TBS composition by adding 10-fold concentrated TBS for stock.

REFERENCES

1. Urayama, O., Murakoshi, T. & Ikawa, Y. K rev-1 protein is abundantly expressed in the rat spinal cord. *Biochem Biophys Acta* **1243**, 446-452 (1995).
2. Kamei, Y. *et al*. FOXO1 activates glutamine synthetase gene in mouse skeletal muscles through a region downstream of 3'-UTR: possible contribution to ammonia detoxification. *Am J Physiol Endocrinol Metab* **307**, E485–E493 (2014).
3. Iwashita, S. *et al*. A transposable element-mediated gene divergence that directly produces a novel type bovine Bcnt protein including the endonuclease domain of RTE-1. *Mol Biol Evol* **20**, 1556-1563 (2003).
4. Kaspersen, J. D., Søndergaard, A., Madsen, D. J., Otzen, D. E. & Pedersen, J. S. Refolding of SDS-unfolded proteins by nonionic surfactants. *Biophys J* **112**, 1609-1620 (2017).
5. Yamakuni, T. *et al*. Isolation and immunohistochemical localization of a cerebellar protein. *Neurosci Lett* **45**, 235-240 (1984).
6. Kurien, B.T. Affinity Purification of Autoantibodies from an Antigen Strip Excised from a Nitrocellulose Protein Blot. *Protein Blotting and Detection. Methods in Molecular Biology (Methods and Protocols)* (eds. Kurien, B., Scofield, R.) vol 536 (Humana Press, Totowa, NJ, 2009).
7. Kulijewicz-Nawrot, M., Syková, E., Chvátalm, A., Verkhratsky, A., & Rodríguez, J. J. Astrocytes and glutamate homoeostasis in Alzheimer's disease: a decrease in glutamine synthetase, but not in glutamate transporter-1, in the prefrontal cortex. *ASN Neuro* **5**, 273-282 (2013).
8. Huyghe, D. *et al*. Glutamine synthetase stability and subcellular distribution in astrocytes are regulated by γ-aminobutyric type B receptors. *J Biol Chem* **289**, 28808-28815 (2014).

LEGENDS OF SUPPLEMENTARY INFORMATION

Table S1: Properties of four types of anti-Bcnt Abs.

This table shows properties of anti-Bcnt Abs and the GYFE fraction from the polyclonal anti-BCNT-C Ab used in this study. "GYIE" of Bcnt, "GYFE" of GS and cysteines for conjugation with keyhole limpet hemocyanin or a resin of affinity-column are indicated in green, red and blue letters, respectively. As for details of preparation of Abs, see Supplementary Materials and Methods online.

Table S2: List of the identified proteins in a 43 kDa band by LC-MS/MS.

The list indicates GI (GenInfo Identifier) number, name, mascot score, number of matched peptides, coverage and exponentially modified protein abundance index (emPAI) of proteins identified by LC-MS/MS from a 43 kDa band. A major protein of the 43 kDa band was GS with a Mascot score of 5925 and emPAI of 68.59

Table S3: Oligonucleotide sequences for construction and confirmation of various plasmids.

Nucleotide sequences of PCR primers used for amplification of mGS and mBcnt cDNA (Table S3-1), construction of deletion or substitution mutants of mGS (Table S3-2), and DNA sequencing (Table S3-3) are listed.

Figure S1: Evaluation of antigen-specificity of isolated anti-BCNT-C Abs.

Antigen-specificity of affinity-purified anti-BCNT-C Abs was evaluated by Western blotting of extracts of rat cerebellum (20 μg/lane) on postnatal day 56 with 10,000 (Nos. 1

and 2)- and 5,000 (No. 3)-fold diluted Abs in the absence (lanes 1, 4, 7) or presence of the BCNT-C-NH_2 peptide (lanes 2, 5, 8) or the Cys-BCNT-C-COOH peptide (lanes 3, 6, 9) used for immunization as an antigen. Data are shown using three representative Abs (Nos. 1, 2, 3) among Abs purified from eight animals. Strong 43/45 kDa bands were detected by all three Abs (lanes 1, 4, 7). Note that these positive bands were completely abolished in the presence of the BCNT-C peptide, regardless of its terminal forms.

Figure S2: The 43 kDa protein as a major reactant in Western blot with anti-BCNT-C Ab.

Western blotting was conducted with the anti-BCNT-C Ab using extracts of bovine brain (Bovine), mouse brain (Mouse), and HEK 293T cells transfected with the expression vector alone (Vector), with the one carrying tag-free mouse *Bcnt*, (mBcnt), or Flag-tagged mouse *Bcnt* (F-mBcnt), as shown on the top of each lane.

Figure S3: Immunoprecipitation of the 43 kDa protein from extracts of bovine brain and rat olfactory bulb.

(A) Comparison of two solubilizing methods for immunoprecipitation. Bovine brain extracts adjusted to the composition of RIPA buffer were mixed with agarose conjugating the anti-BCNT-C Ab (α-BCNT) or with its denatured agarose (Control). Bound or unbound fractions of each mixture were subjected to Western blotting with the anti-BCNT-C Ab (1 μg/mL, left panel). The extracts boiling in SDS followed by dilution with the HBS containing 0.1% NP40 were treated in the same manner and detected (right panel). “Total” and “p43” at the top of the column denote the starting extracts for immunoprecipitation and

the isolated 43 kDa protein shown in Fig. 2, respectively. ***(B)*** Immunoprecipitation of the 43 kDa protein from extracts of rat olfactory bulb. Extracts of rat olfactory bulb [1] were treated in SDS and subjected to immunoprecipitation with the anti-BCNT-C Ab by the same procedures as shown in *(A)*.

Figure S4: Isolation of the 43 kDa protein from bovine brain extracts.

Bovine brain extracts were fractionated by ammonium sulfate precipitation, and the pellet was applied to a phenyl-sepaharose column (see supplementary Materials and Methods). ***(A)*** The bound proteins were eluted with an n-octyl glucoside-containing solution. Numbers 1–5 on each lane indicate the order of eluted fractions, and 6 shows the irreversibly adsorbed proteins recovered by boiling the resin in SDS-PAGE sample buffer. ***(B)*** Three fractions showing the most intense immunoreactivity with the anti-BCNT-C Ab (shown in red half bracket in upper panel of *A*) were pooled and subjected to acetone precipitation. The resuspended protein was boiled in SDS and subjected to immunoprecipitation with an anti-BCNT-C Ab-linked agarose, and the bound proteins were eluted with the antigen peptides. Lane 1: fraction unbound to the resin, lanes 2 and 3: wash fractions, lanes 4 and 5: eluted fractions, and lane 6: non-eluted fraction recovered as described above. Lower panels of A and B are CBB staining of the membranes shown in the upper panels after Western blotting by the colorimetric method.

Figure S5: Identification of the 43 kDa protein as GS by LC-MS/MS.

The three CBB-positive bands detected by SDS-PAGE shown in Fig. 2 were analyzed after digestion with trypsin. ***(A)***; Resulting fragments recovered from the 43 kDa band cover

76.9% of amino acid residues of GS (shown in red bold letters). The 49 amino acid residues with a green underline indicate a fragment with no trypsin cleavage sites. ***(B)***; A representative MSMS spectrum for the identification of GS is shown. The peptide VQAMYIWIDGTGEGLR derived from the 43 kDa band, corresponding to a red underline shown in Fig. S5A, was identified as GS with a score of 99 and an expect value of 3e-008 using Mascot (see also Supplementary Table S1).

Figure S6: Recognition of GS derived from two sources by the anti-BCNT-C Ab.

Western blotting of purified recombinant human GS (Rec GS) with a 20-amino acid His-tag at the N-terminal (left panel) and of extracts of skeletal muscles of wild-type or a FOXO-1 transgenic mouse (FOXO-1 Tg, right panel), respectively with either anti-BCNT-C Ab (1 μg/mL) or anti-GS Ab (1 μg/mL) by the colorimetric method. Extracts of mouse brain were used as a positive control in both panels.

Figure S7: Detection of immunoreactivity by the anti-BCNT-C Ab in astrocytes but not neurons of rat cerebellum.

Green and red fluorescence indicate the immunoreactivities with the anti-BCNT-C Ab (A, D and G), anti-GS Ab (B), anti-GFAP Ab (E), or anti-Spot 35 (Calbindin-D28K) Ab (H), respectively. C, F and I are merged figures of both fluorescences. Blue fluorescence indicates nuclear staining by TO-PRO-3. GFAP and Spot35 are used as a marker of astrocytes and Purkinje cells, respectively. Note that the immunoreactivity by the anti-BCNT-C Ab is colocalized with GS- and GFAP-immunoreactivity, but not with Spot 35-immunoreactivity. Scale bars indicate 50 μm. Abbreviation: Mol, molecular layer; Pur,

Purkinje cell layer; Gr, granular layer.

Figure S8: Similar characteristics of the 43 kDa protein to GS.

(A) Expression changes of the 43 kDa protein during postnatal developmental of rat brain. Extracts of rat cerebrum at each postnatal day (denoted by P# on the top of each lane, 3 μg per lane) were loaded and subjected to Western blotting with the anti-BCNT-C Ab. The same filter was reprobed with an anti-β-actin Ab (250 ng/mL, lower panel). Both signals were detected by the chemiluminescent method. The expression profile is consistent with that of mouse GS in the brain previously reported [7]. (B) Intracellular localization of the 43 kDa protein. Cellular components of adult rat whole cerebrum were fractionated using a subcellular fractionation kit (Invitrogen). Each fraction is shown at the top of each lane: cytosol, cell membrane/organelle, nuclei, and cytoskeleton, respectively. The samples with equivalent ratios of each fraction volume were subjected to Western blotting with the anti-BCNT-C Ab (50 ng/mL). Whole and Mix denote the extracts before cellular fractionation and mixtures of each fraction, respectively. The result of preferential membrane localization of the immunoreactivity is consistent with a report that GS is associated with GABA receptors in the plasma membrane of rat astrocytes [8].

Figure S9: GS peptides for competition experiments and concentration-dependent inhibition of the anti-BCNT-C Ab immunoreactivity by the P1 peptide.

Upper panel and lower table show arrangements and information of amino acid sequences of four peptide fragments (P1–P4) used for competition experiments, respectively. The 346th and 359th cysteine residues of GS (red letters) were replaced with serine as shown in the

lower table. Middle panel shows Western blots of *E. coli* extracts expressing full-length GS (5 μg per lane) with the anti-BCNT-C Ab (1 μg/mL) preincubated with various concentration (μM, indicated by figures on each lane) of peptide P1. None means a negative control containing DMSO only. Another negative control was 100 μM peptide P2 (rightmost lane). Western blot signals were detected by the colorimetric method.

Figure S10: Evaluation of the affinity of the anti-BCNT-C Ab to GS and BCNT.

Comparison of the anti-BCNT-C Ab reactivity against mGS and mBcnt. Extracts of *E. coli* expressing Flag-mGS or Flag-mBcnt, which were adjusted to show an equal immunoreactivity using anti-Flag Ab, were subjected to Western blotting. The membrane was first reacted with anti-Flag Ab to confirm loading amounts. After stripping, the membrane was reprobed with untreated anti-BCNT-C Ab (Whole) or affinity-purified Abs from membrane bound to wild-type GS (WT) or All-Ala GS mutant (All-Ala, see Fig. 4D). The asterisk indicates non-specific band.

Figure S11: Full-length images of figures shown as cropped forms in the main text of the manuscript.

A, **B**, **C**, **D**, **E**, **F**, **G** and **H** are full-length images of Fig. 3A, 4A, 4B, 4C, 4D, 5A, 5B and 6A, respectively, which are shown as cropped forms in the main text of the manuscript.

Table S1

Properties of four types of anti-Bcnt Abs

Ab or Fraction name	Immunized animal	Property
Polyclonal anti-**BCNT-C** Ab	Guinea pig	An Ab raised against a peptide derived from C-terminal region of Bcnt.
	Sequence of the antigen peptide NH_2-**CEELAIHNRGKEGYIERKA**-COOH	
Polyclonal anti-**Bcnt-Cter** Ab	Guinea pig	An Ab raised against a peptide derived from C-terminus of Bcnt, which is different from the antigen peptide for the polyclonal anti-BCNT-C Ab.
	Sequence of the antigen peptide NH_2-**CRDLRLSKMKP**-COOH	
Polyclonal anti-**mBcnt-N** Ab	Guinea pig	An Ab raised against a peptide derived from N-terminal region of mouse Bcnt, which is different from the antigen peptide for the polyclonal anti-BCNT-C Ab.
	Sequence of the antigen peptide CH_3CO-**GEEQAEKTKGKRRKAQC**-COOH	
**GYFE fraction** of the polyclonal anti-BCNT-C Ab	A subpopulation of the polyclonal anti-BCNT-C Ab cross-reacting with "GYFE" of GS, which is isolated by affinity-column using GS (332-346) peptide.	
	Sequence of GS(332-346) peptide CH_3CO-**EKKGYFEDRRPSANC**-COOH	

Table S2

List of the identified proteins in 43 kDa band by LC-MS/MS

GI number	Protein name	Mascot Score	Number of matched peptides	Coverage (%)	emPAI
gi|95006991	glutamine synthetase [Bos taurus]	5,925	31	76.9	68.59
gi|75812932	actin, cytoplasmic 2 [Bos taurus]	1,401	18	58.4	5.55
gi|27819614	actin, alpha skeletal muscle [Bos taurus]	700	12	33.4	1.93
gi|741897519	PREDICTED: beta-actin-like protein 2 [Bos taurus]	425	10	38.0	1.21
gi|168804008	tubulin alpha-1B chain [Bos taurus]	96	3	8.4	0.20
gi|262073106	cathepsin D precursor [Bos taurus]	82	2	6.3	0.15
gi|28849933	craniofacial development protein 1 [Bos taurus]	62	2	2.7	0.20

Table S3-1

PCR primers for amplification of mGS and mBcnt cDNA

No.	Name	Sequence		
**mGS-Fw**	Full-mGS-pCold_Fw	5'-	GCCGCATATGGCCACCTCAGCAAG	-3'
**mGS-Rev**	Full-mGS-pCold_Rev	5'-	GCCGCTCGAGTTAGTTCTTGTATTGGAAGG	-3'
**mGS2-Fw**	NheI-BglII-koz-mGS_Fw	5'-	GCTAGCAGATCTGCCGCCATGGCCACCTCAGCAAGTTC	-3'
**mGS2-Rev**	mGS-XhoI-XbaI_Rev	5'-	TCTAGACTCGAGTTAGTTCTTGTATTGGAAGGGTTCGTC	-3'
**F-mGS-Fw**	NheI-BglII-mGS_Fw	5'-	GCTAGCAGATCTATGGCCACCTCAGCAAGTTC	-3'
**F-mGS-Rev**	mGS-XhoI-XbaI_Rev	5'-	TCTAGACTCGAGTTAGTTCTTGTATTGGAAGGGTTCGTC	-3'
**mBcnt-Fw**	SacI-BamHI-kozak-mbcnt Fw	5'-	GAGCTCGGATCCGCCGCCATGGAGGAATTCGACTCCGAAG	-3'
**mBcnt-Rev**	mbcnt-XhoI-XbaI_Rev	5'-	TCTAGACTCGAGTCAAGGTTTCATTTTGCTCAGCCTG	-3'
**F-mBcnt-Fw**	NheI-BamHI-mbcnt_Fw	5'-	GCTAGCGGATCCATGGAGGAATTCGACTCCGAAGAC	-3'
**F-mBcnt-Rev**	mbcnt-XhoI-XbaI_Rev	5'-	TCTAGACTCGAGTCAAGGTTTCATTTTGCTCAGCCTG	-3'

Table S3-2

PCR primers for GS mutant construction

No.	Name	Sequence		
**1-Fw**	Del(1-197)_mGS-pCold_Fw	5'-	GCCGCATATGCCTGCCCAGTGGGA	-3'
**1-Rev**	Del(1-197)_mGS-pCold_Rev	5'-	GCCGCTCGAGTTAGTTCTTGTATTGGAAGG	-3'
**2-Fw**	Del(332-373)_mGS-pCold_Fw	5'-	GTCGGCCAGTAACTCGAGGGATCCGAATTC	-3'
**2-Rev**	Del(332-373)_mGS-pCold_Rev	5'-	CTCGAGTTACTGGCCGACAGTCCGGGGAATG	-3'
**3-Fw**	Del(335-373)_mGS-pCold_Fw	5'-	CAGGAGAAGAAGTAACTCGAGGGATCCGAATTC	-3'
**3-Rev**	Del(335-373)_mGS-pCold_Rev	5'-	CCTCGAGTTACTTCTTCTCCTGGCCGAC	-3'
**4-Fw**	Del(337-373)_mGS-pCold_Fw	5'-	GAAGGGCTACTAACTCGAGGGATCCGAATTC	-3'
**4-Rev**	Del(337-373)_mGS-pCold_Rev	5'-	CCTCGAGTTAGTAGCCCTTCTTCTCCTGG	-3'
**5-Fw**	Del(339-373)_mGS-pCold_Fw	5'-	GGCTACTTTGAATAACTCGAGGGATCCGAATTC	-3'
**5-Rev**	Del(339-373)_mGS-pCold_Rev	5'-	CCTCGAGTTATTCAAAGTAGCCCTTCTTCTCC	-3'
**6-Fw**	Del(340-373)_mGS-pCold_Fw	5'-	CTTTGAAGACTAACTCGAGGGATCCGAATTC	-3'
**6-Rev**	Del(340-373)_mGS-pCold_Rev	5'-	CCTCGAGTTAGTCTTCAAAGTAGCCCTTCTTC	-3'
**7-Fw**	Del(346-373)_mGS-pCold_Fw	5'-	CTGCCAATTAACTCGAGGGATCCGAATTC	-3'
**7-Rev**	Del(346-373)_mGS-pCold_Rev	5'-	CTCGAGTTAATTGGCAGAAGGCCGACG	-3'
**8-Fw**	Ala(G335A)_mGS-pCold_Fw	5'-	GAGAAGAAGGCCTACTTTGAAGACCGTCGGCCTTC	-3'
**8-Rev**	Ala(G335A)_mGS-pCold_Rev	5'-	TTCAAAGTAGGCCTTCTTCTCCTGGCCGACAG	-3'
**9-Fw**	Ala(Y336A)_mGS-pCold_Fw	5'-	AAGGGCGCCTTTGAAGACCGTCGGCCTTC	-3'
**9-Rev**	Ala(Y336A)_mGS-pCold_Rev	5'-	TCAAAGGCGCCCTTCTTCTCCTGGCC	-3'
**10-Fw**	Ala(F337A)_mGS-pCold_Fw	5'-	AGGGCTACGCAGAAGACCGTCGGCCTTCTG	-3'
**10-Rev**	Ala(F337A)_mGS-pCold_Rev	5'-	GTCTTCTGCGTAGCCCTTCTTCTCCTGGC	-3'
**11-Fw**	Ala(E338A)_mGS-pCold_Fw	5'-	GCTACTTTGCAGACCGTCGGCCTTCTGC	-3'
**11-Rev**	Ala(E338A)_mGS-pCold_Rev	5'-	GACGGTCTGCAAAGTAGCCCTTCTTCTCCTGG	-3'
**12-Fw**	All-Ala(335ALL338)_mGS-pCold_Fw	5'-	GGCCGCCGCAGCAGACCGTCGGCCTTCTGCCAATTG	-3'
**12-Rev**	All-Ala(335ALL338)_mGS-pCold_Rev	5'-	CTGCTGCGGCGGCCTTCTTCTCCTGGCCGACAGTCCG	-3'
**13-Fw**	A4(AAGYFEAA)_mGS-pCold_Fw	5'-	GCAGCAGGCTACTTTGAAGCCGCACGGCCTTCTGCCAATTGTGACCCCTATGC	-3'
**13-Rev**	A4(AAGYFEAA)_mGS-pCold_Rev	5'-	TGCGGCTTCAAAGTAGCCTGCTGCCTCCTGGCCGACAGTCCGGGGAATG	-3'
**14-Fw**	Ile(F337I)_mGS-pCold_Fw	5'-	AGGGCTACATTGAAGACCGTCGGCCTTC	-3'
**14-Rev**	Ile(F337I)_mGS-pCold_Rev	5'-	GGTCTTCAATGTAGCCCTTCTTCTCCTGGC	-3'
**15-Fw**	Leu(F337L)_mGS-pCold_Fw	5'-	GGCTACCTGGAAGACCGTCGGCCTTCTG	-3'
**15-Rev**	Leu(F337L)_mGS-pCold_Rev	5'-	GGTCTTCCAGGTAGCCCTTCTTCTCCTGGC	-3'
**16-Fw**	Val(F337V)_mGS-pCold_Fw	5'-	GGCTACGTTGAAGACCGTCGGCCTTCTG	-3'
**16-Rev**	Val(F337V)_mGS-pCold_Rev	5'-	GGTCTTCAACGTAGCCCTTCTTCTCCTGGC	-3'
**17-Fw**	Tyr(F337Y)_mGS-pCold_Fw	5'-	GGCTACTATGAAGACCGTCGGCCTTCTG	-3'
**17-Rev**	Tyr(F337Y)_mGS-pCold_Rev	5'-	GGTCTTCATAGTAGCCCTTCTTCTCCTGGC	-3'

Table S3-3

Primers for Sequencing

Name	Sequence		
pCold_Fw-2	5'-	GCTCTCCCTTATGCGACTCC	-3'
pCold_Rev-2	5'-	TCCGCTTACAGACAAGCTGTG	-3'
mGS(100-120)_Fw	5'-	GATGGTACCGGAGAAGGACTG	-3'

Figure S1

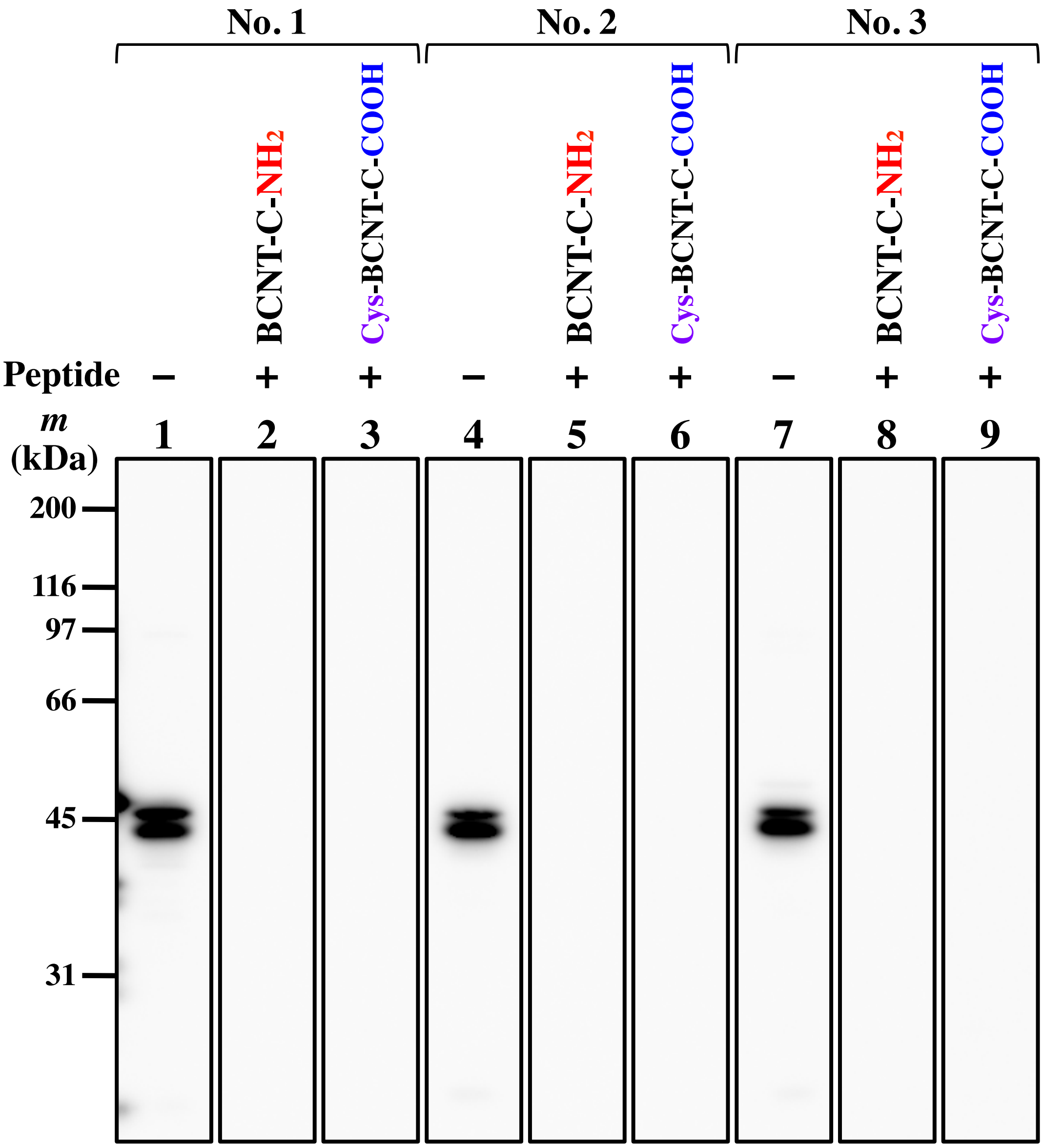
No. 1
No. 2
No. 3
BCNT-C-NH2
Cys-BCNT-C-COOH
Peptide
−
+
m (kDa)
1 2 3 4 5 6 7 8 9
200
116
97
66
45
31

Figure S2

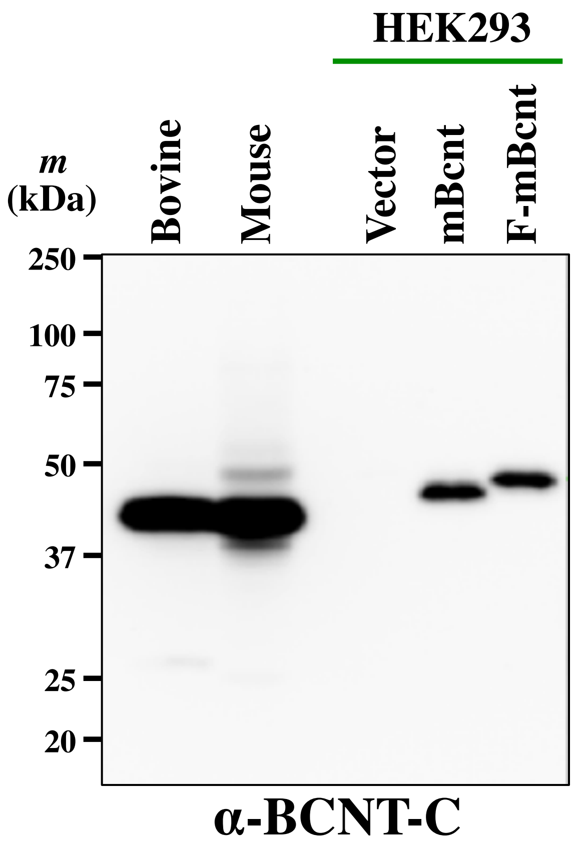
HEK293
m (kDa)
Bovine
Mouse
Vector
mBcnt
F-mBcnt
250
100
75
50
37
25
20
α-BCNT-C

Figure S3

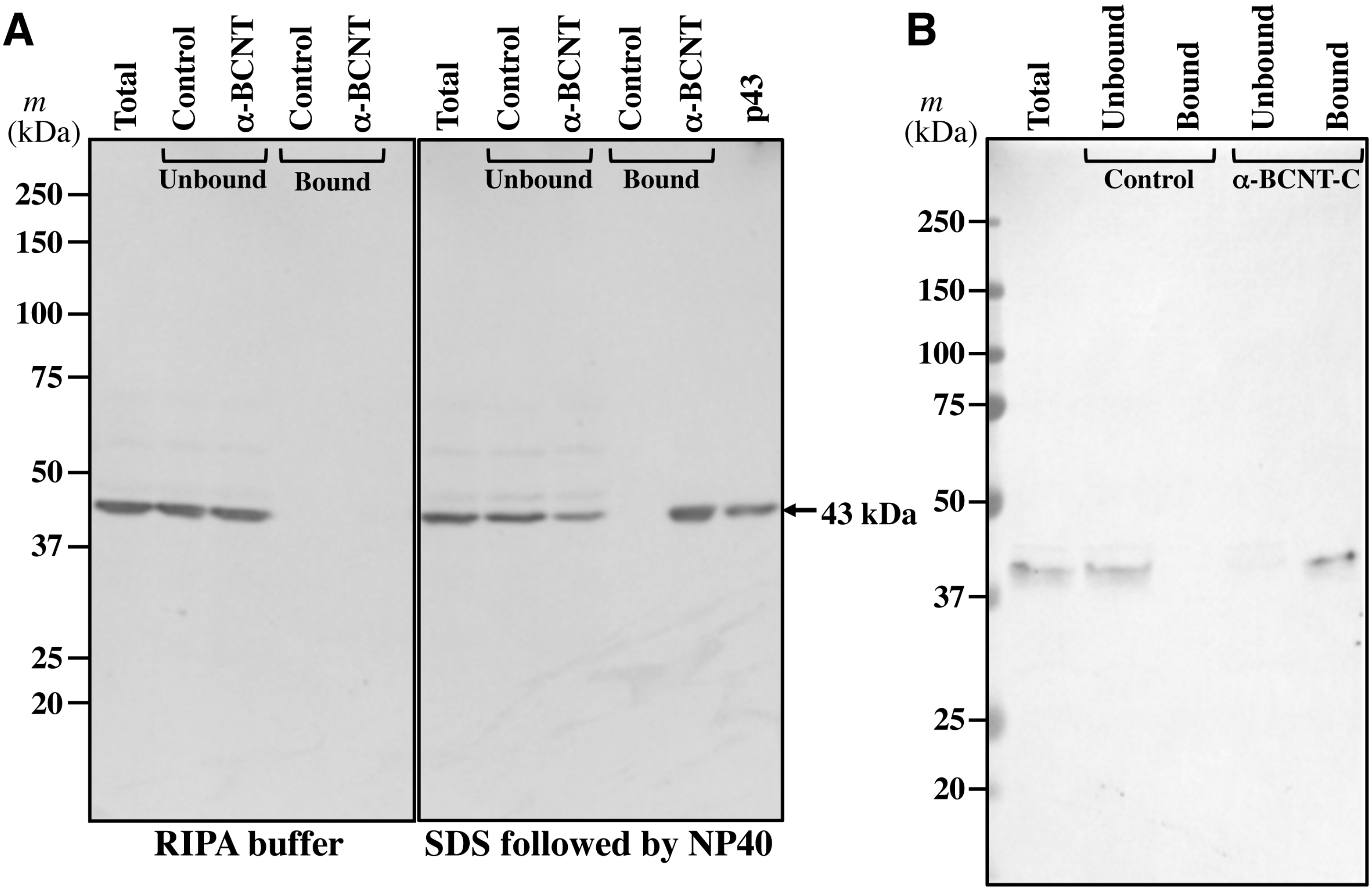
A
m (kDa)
Total
Control
α-BCNT
Control
α-BCNT
Unbound
Bound
250
150
100
75
50
37
25
20
Total
Control
α-BCNT
Control
α-BCNT
p43
Unbound
Bound
43 kDa
RIPA buffer
SDS followed by NP40
B
m (kDa)
Total
Unbound
Bound
Unbound
Bound
Control
α-BCNT-C
250
150
100
75
50
37
25
20

Figure S4

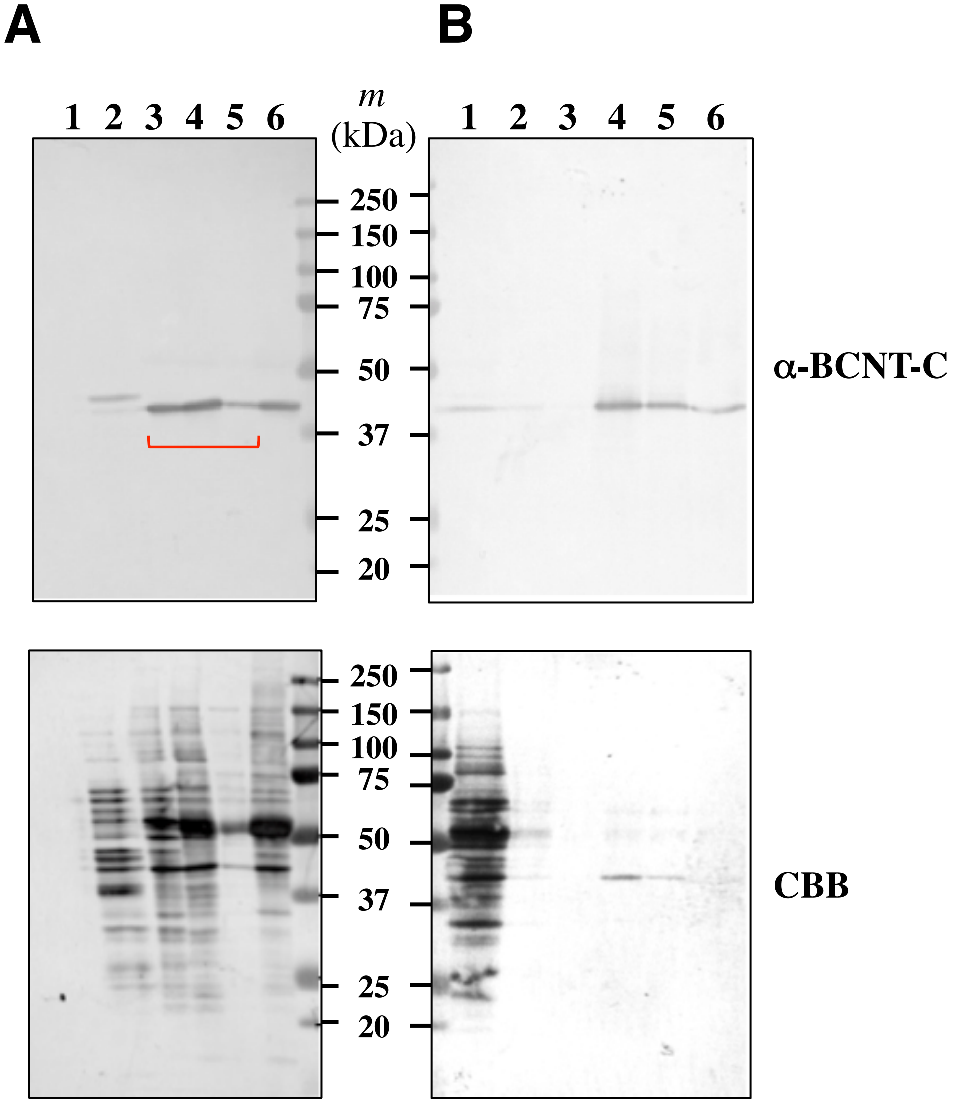

Figure S5

A

```
  1 MATSASSHLN KGIKQVYMAL PQGDKVQAMY IWIDGTGEGL RCKTRTLDSE PKCIEELPEW
 61 NFDGSSTFQS EGSNSDMYLV PAAMFRDPFR KDPNKLVFCE VFKYNRKPAE TNLRHTCKRI
121 MDMVSNQRPW FGMEQEYTLM GTDGHPFGWP SNGFPGPQGP YYCGVGADKA YGRDIVEAHY
181 RACLYAGIKI GGTNAEVMPA QWEFQIGPCE GIDMGDHLWV ARFILHRVCE DFGVIATFDP
241 KPIPGNWNGA GCHTNFSTKA MREENGLKYI EEAIEKLSKR HQYHIRAYDP KGGLDNARRL
301 TGFHETSNIN DFSAGVANRG ASIRIPRTVG QEKKGYFEDR RPSANCDPFA VTEALIRTCL
361 LNETGDEPFQ YKN
```

B

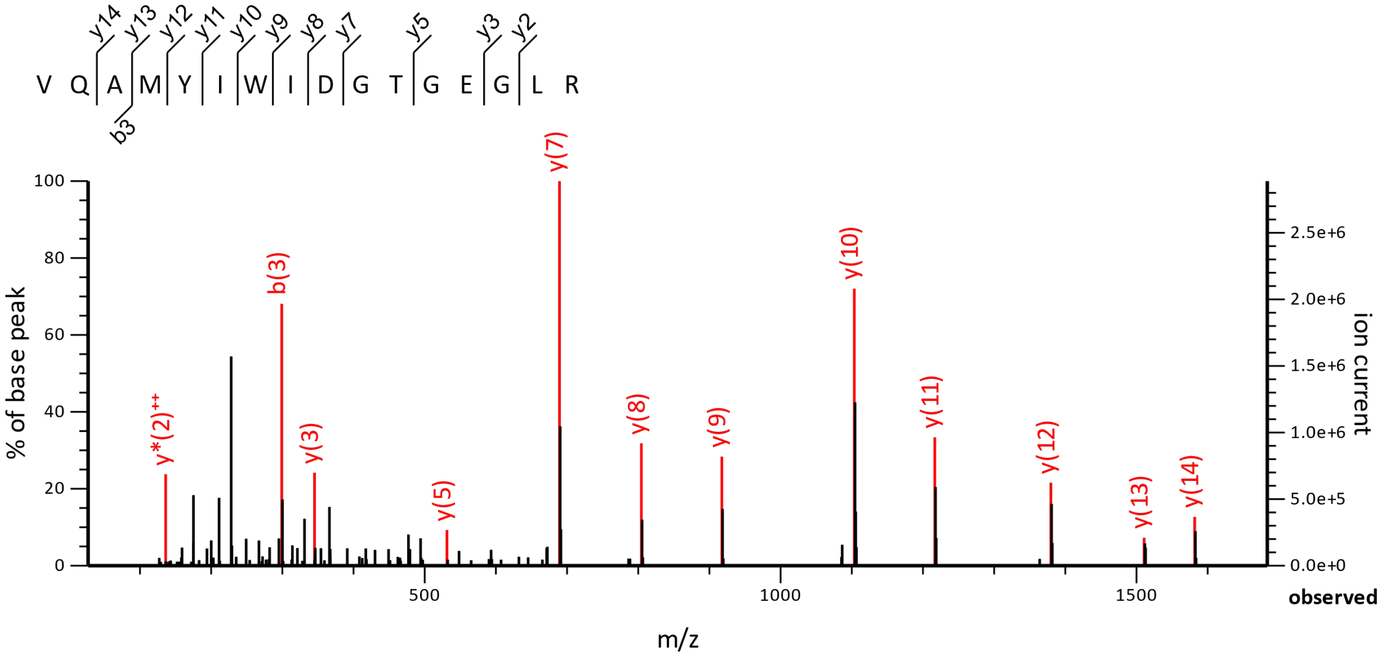

Figure S6

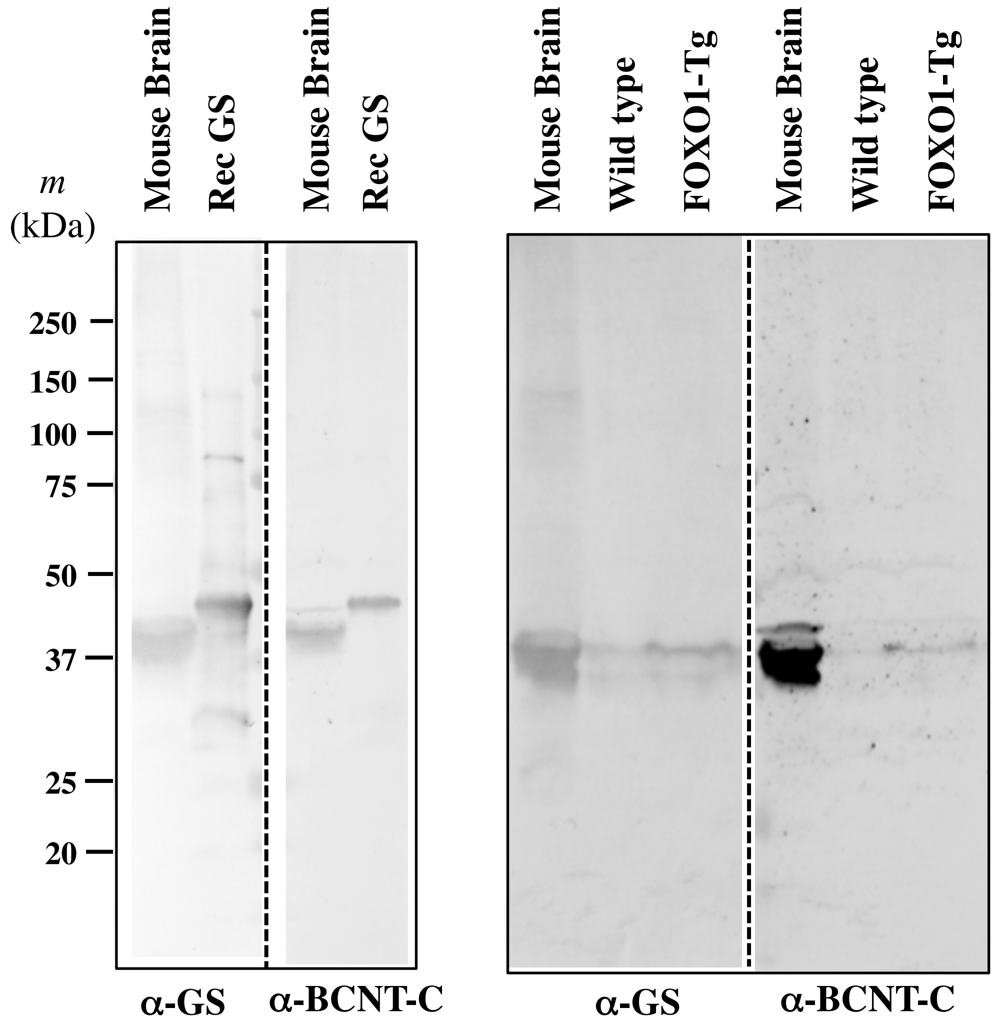
Mouse Brain
Rec GS
Mouse Brain
Rec GS
Mouse Brain
Wild type
FOXO1-Tg
Mouse Brain
Wild type
FOXO1-Tg
m
(kDa)
250
150
100
75
50
37
25
20
α-GS
α-BCNT-C
α-GS
α-BCNT-C

Figure S7

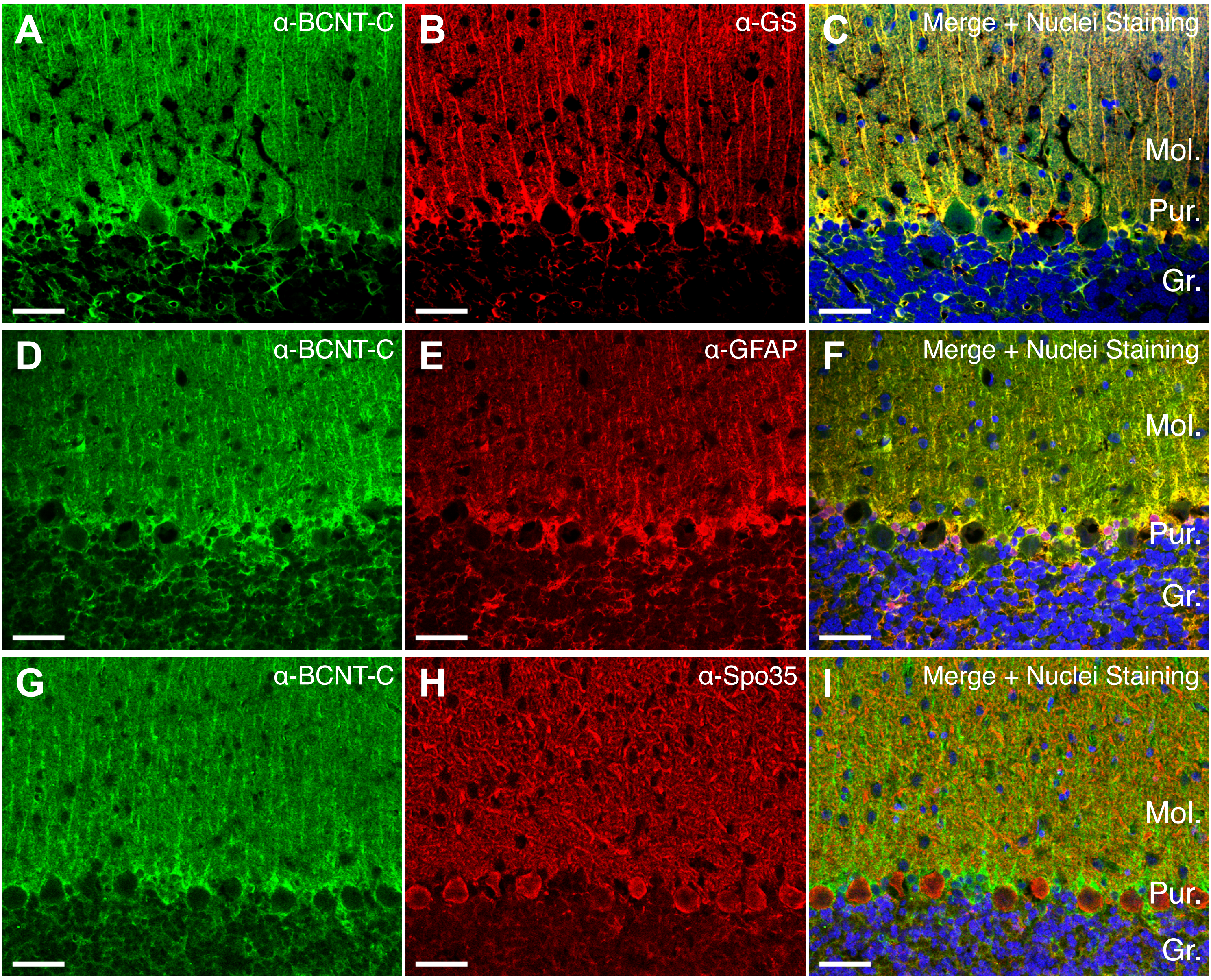
A
α-BCNT-C
B
α-GS
C
Merge + Nuclei Staining
Mol.
Pur.
Gr.
D
α-BCNT-C
E
α-GFAP
F
Merge + Nuclei Staining
Mol.
Pur.
Gr.
G
α-BCNT-C
H
α-Spo35
I
Merge + Nuclei Staining
Mol.
Pur.
Gr.

Figure S8

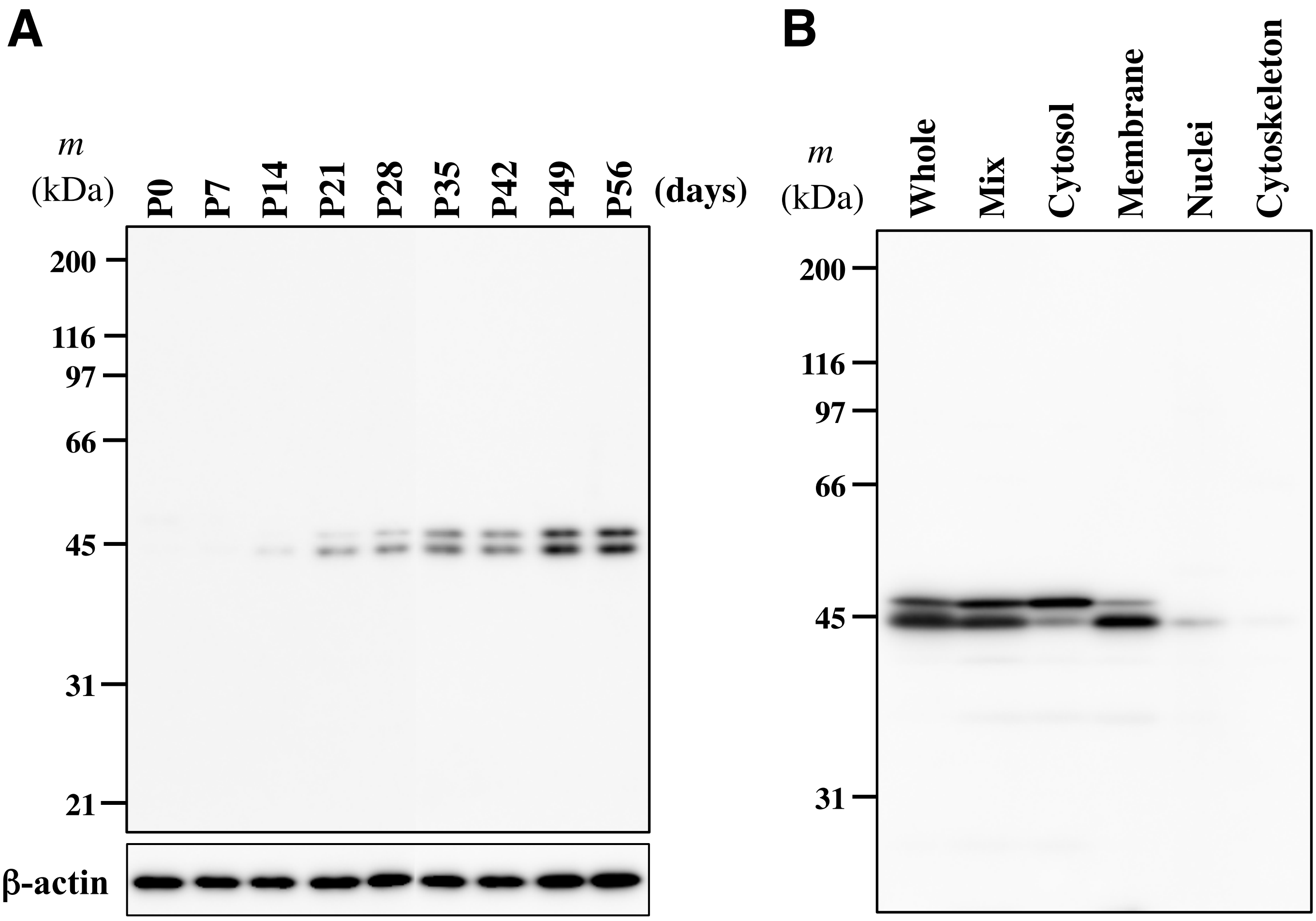

A
m
(kDa)
P0
P7
P14
P21
P28
P35
P42
P49
P56
(days)
200
116
97
66
45
31
21
β-actin
B
m
(kDa)
Whole
Mix
Cytosol
Membrane
Nuclei
Cytoskeleton
200
116
97
66
45
31

Figure S9

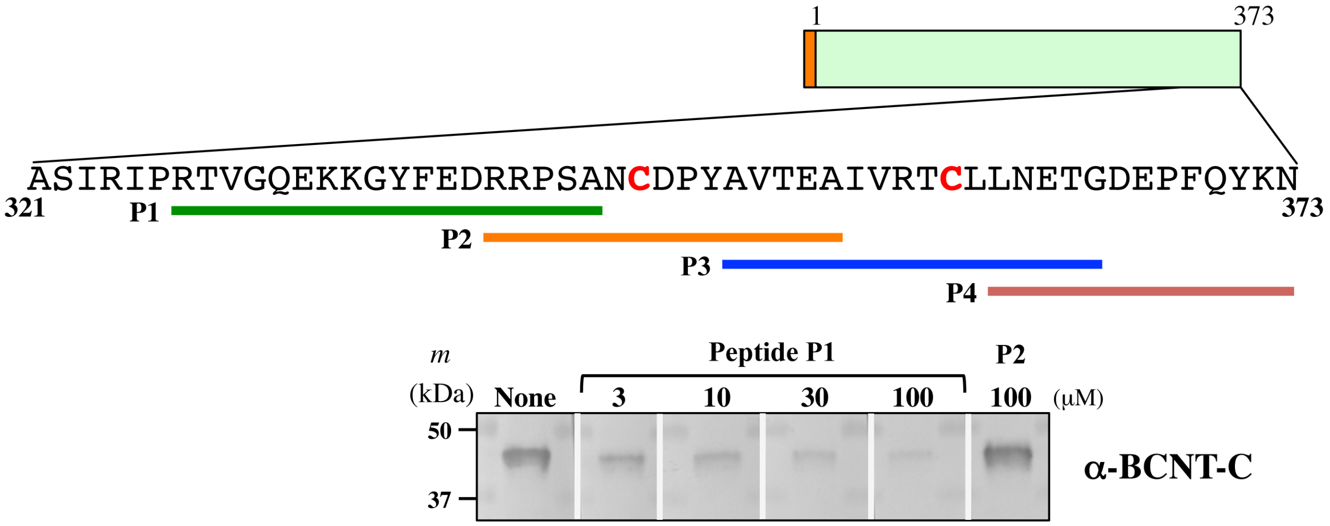

Peptide Name	Amino acid Sequence	Molecular weight	Purity (%)
P1	Acetyl-RTVGQEKKGYFEDRRPSA-NH2	2165.38	71.3
P2	Acetyl-RRPSANSDPYAVTEA-NH2	1674.79	75.1
P3	Acetyl-AVTEAIVRTSLLNETG-NH2	1714.90	88.9
P4	Acetyl-LNETGDEPFQYKN-OH	1596.65	75.5

Figure S10

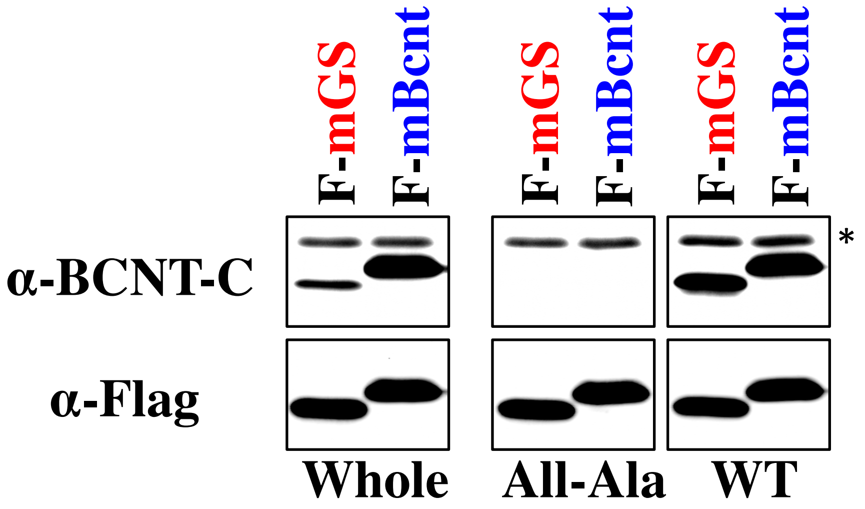
F-mGS
F-mBcnt
F-mGS
F-mBcnt
F-mGS
F-mBcnt
α-BCNT-C
α-Flag
*
Whole
All-Ala
WT

Figure S11

A

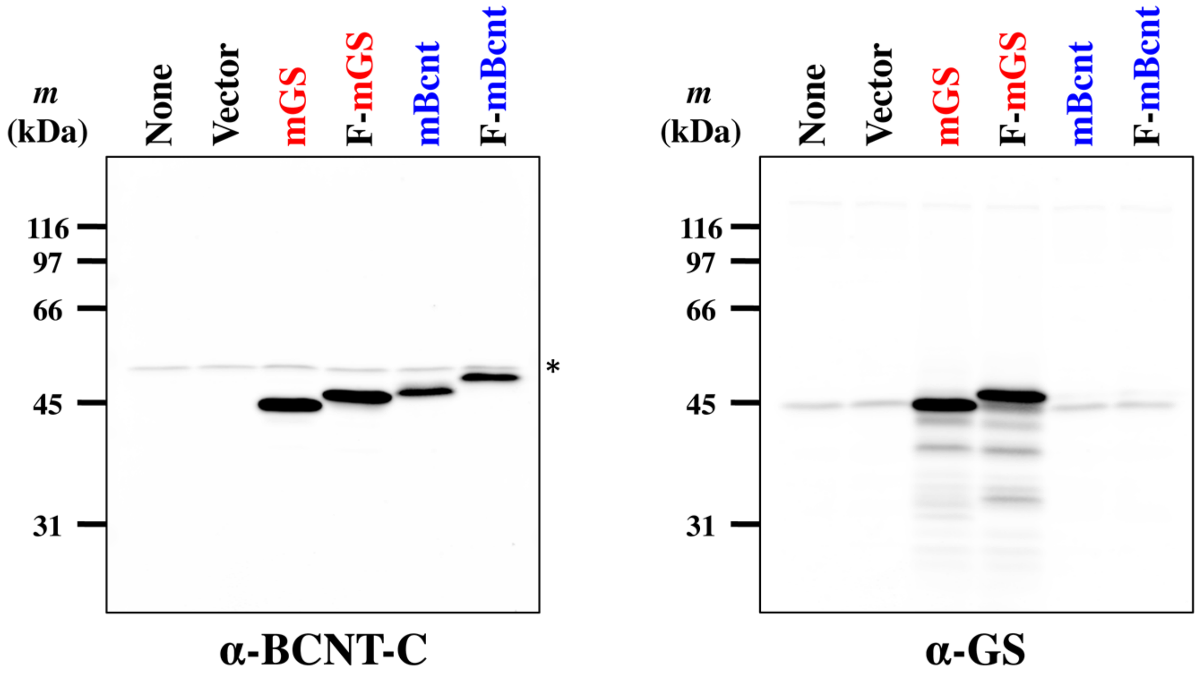

B

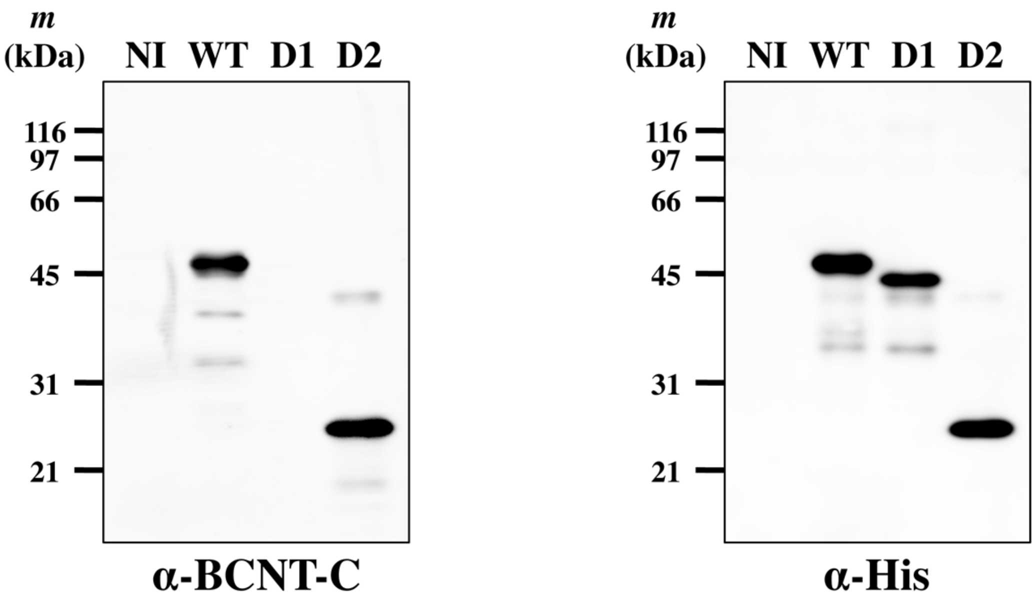

Figure S11

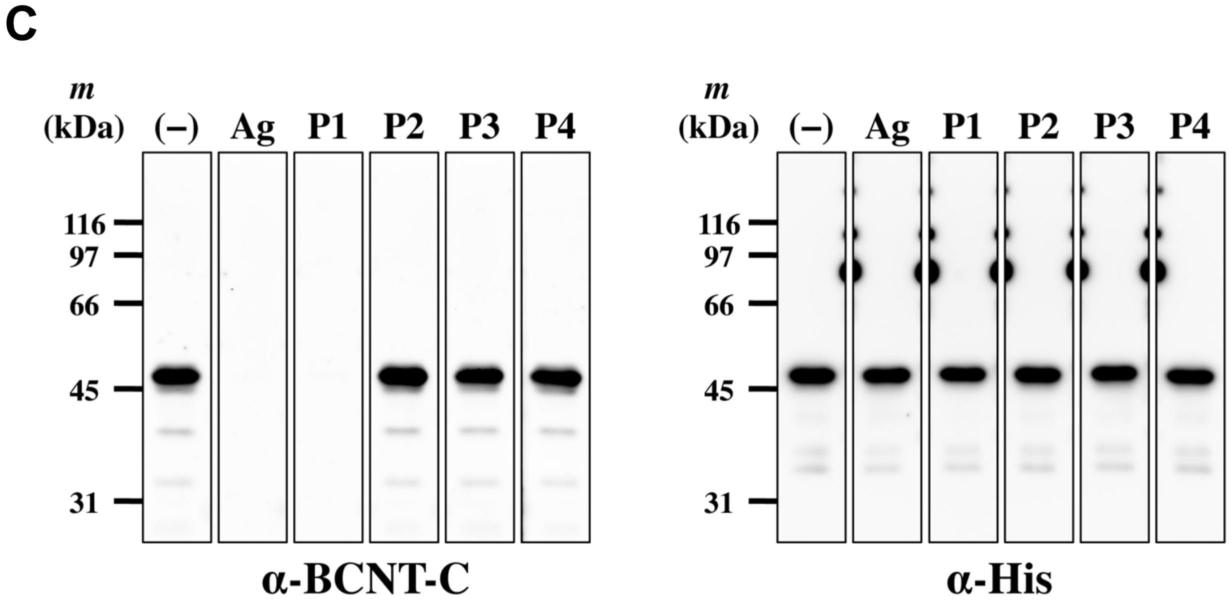
C
m
(kDa)
(–)
Ag
P1
P2
P3
P4
116
97
66
45
31
α-BCNT-C
α-His

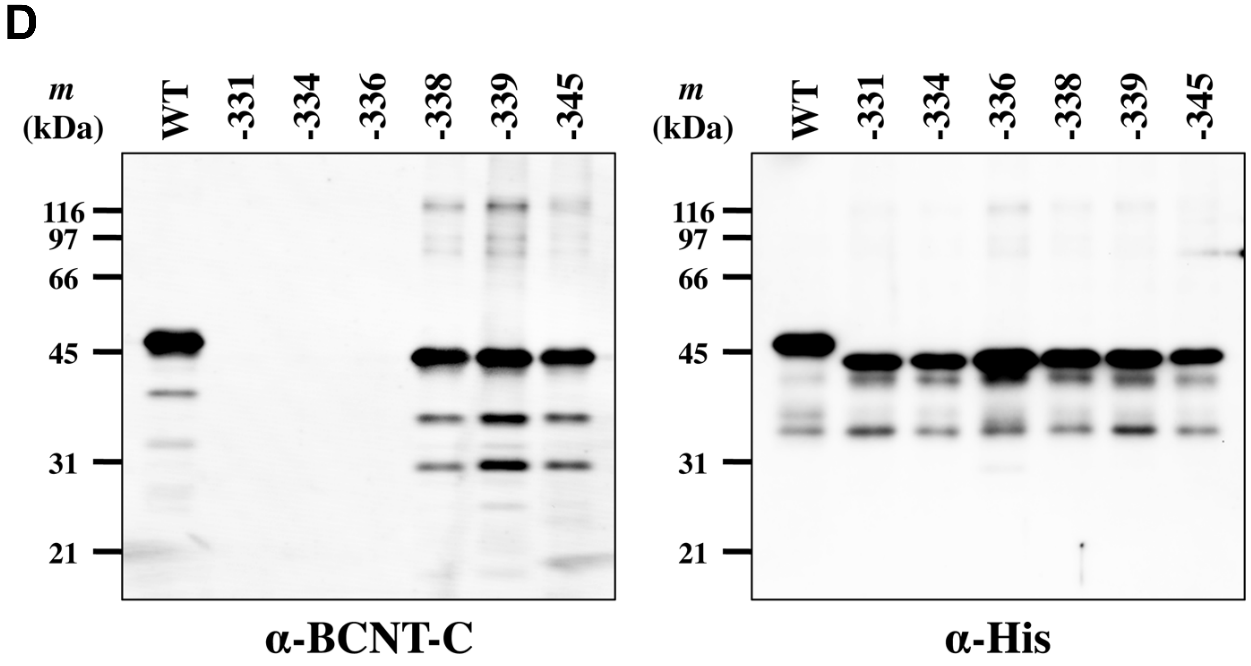
D
m
(kDa)
WT
-331
-334
-336
-338
-339
-345
116
97
66
45
31
21
α-BCNT-C
α-His

Figure S11

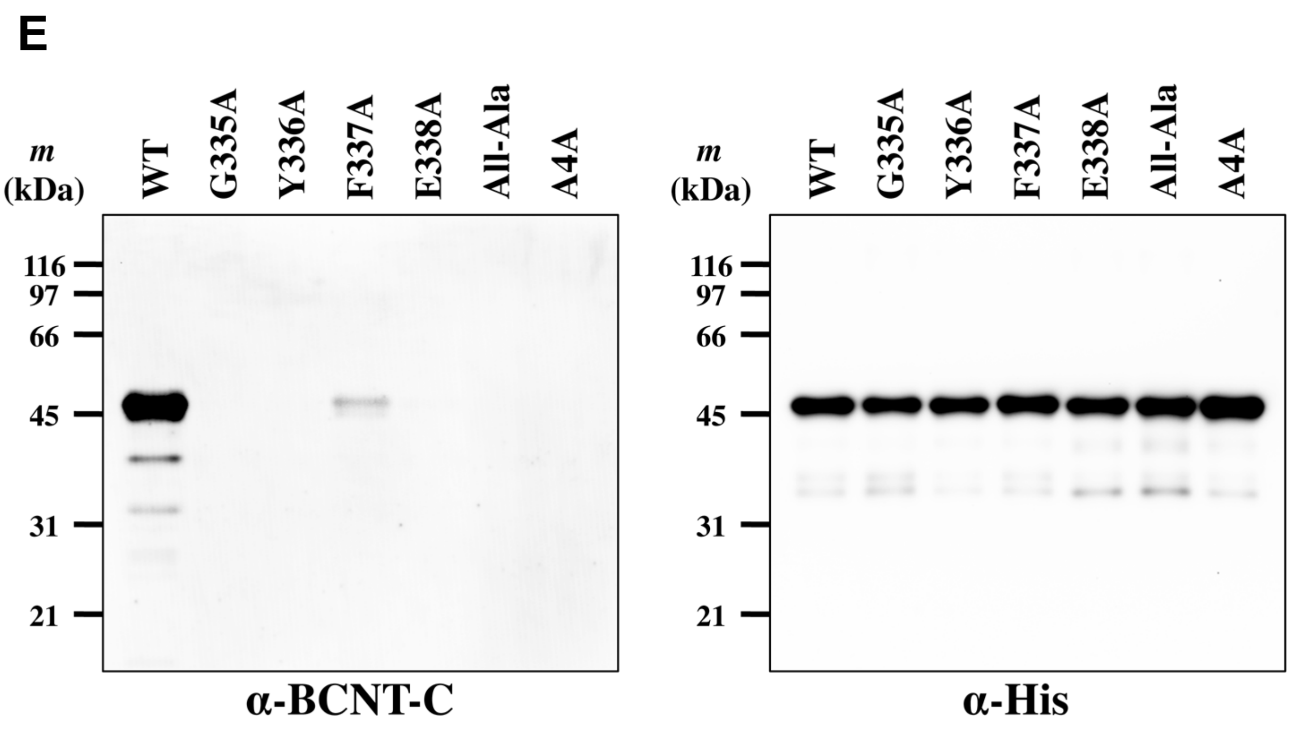
E
m (kDa)
WT
G335A
Y336A
F337A
E338A
All-Ala
A4A
116
97
66
45
31
21
α-BCNT-C
α-His

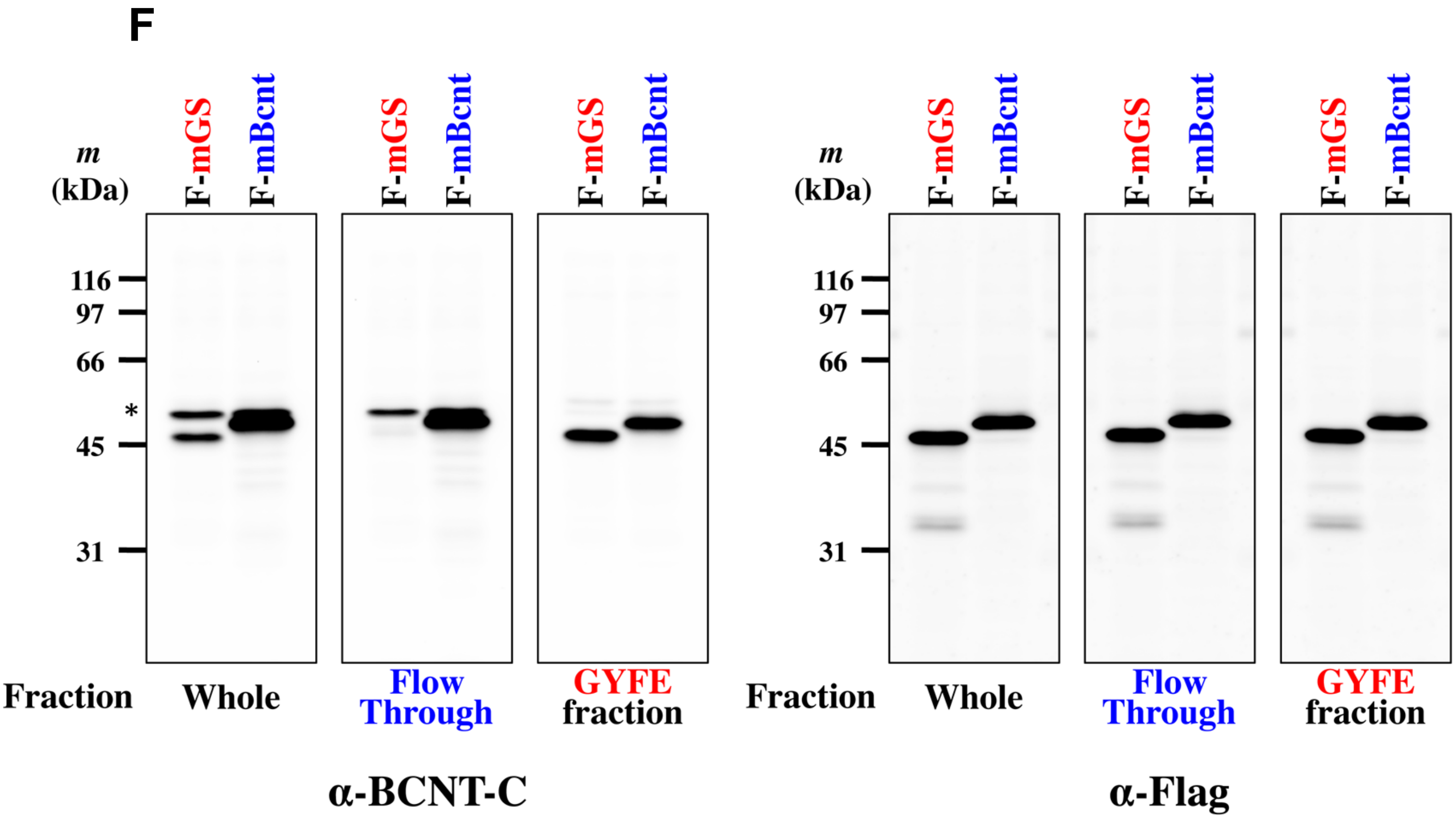
F
m (kDa)
F-mGS
F-mBcnt
116
97
66
*
45
31
Fraction
Whole
Flow Through
GYFE fraction
α-BCNT-C
α-Flag

Figure S11

G

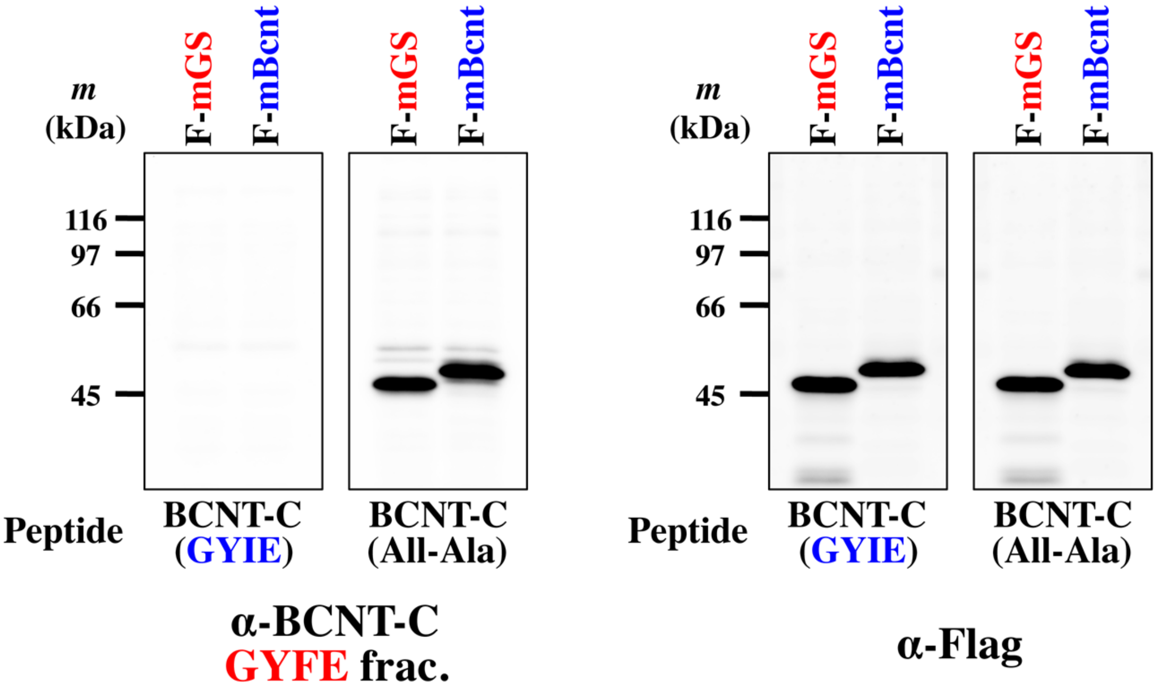

H

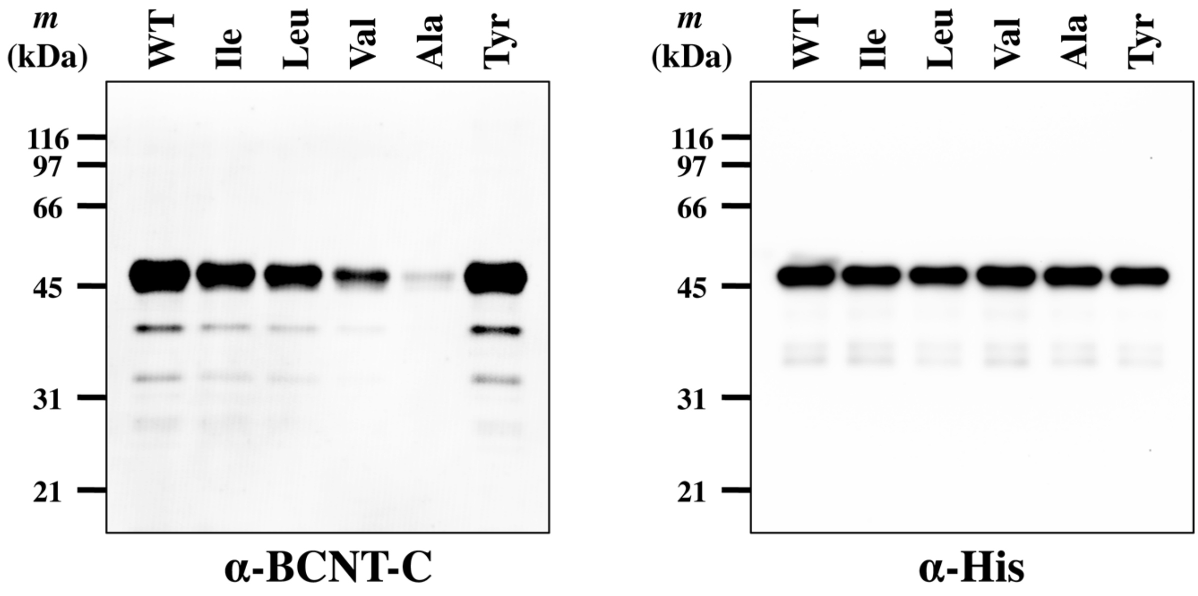

Supplement: Supplementary file 1 — Supplementary Information [file 41598_2019_51350_MOESM1_ESM.pdf]
